# Atg6/UVRAG/Vps34-Containing Lipid Kinase Complex Is Required for Receptor Downregulation through Endolysosomal Degradation and Epithelial Polarity during *Drosophila* Wing Development

**DOI:** 10.1155/2014/851349

**Published:** 2014-05-21

**Authors:** Péter Lőrincz, Zsolt Lakatos, Tamás Maruzs, Zsuzsanna Szatmári, Viktor Kis, Miklós Sass

**Affiliations:** Department of Anatomy, Cell and Developmental Biology, Eotvos Lorand University, Budapest 1117, Hungary

## Abstract

Atg6 (Beclin 1 in mammals) is a core component of the Vps34 PI3K (III) complex, which promotes multiple vesicle trafficking pathways. Atg6 and Vps34 form two distinct PI3K (III) complexes in yeast and mammalian cells, either with Atg14 or with UVRAG. The functions of these two complexes are not entirely clear, as both Atg14 and UVRAG have been suggested to regulate both endocytosis and autophagy. In this study, we performed a microscopic analysis of UVRAG, Atg14, or Atg6 loss-of-function cells in the developing *Drosophila* wing. Both autophagy and endocytosis are seriously impaired and defective endolysosomes accumulate upon loss of Atg6. We show that Atg6 is required for the downregulation of Notch and Wingless signaling pathways; thus it is essential for normal wing development. Moreover, the loss of Atg6 impairs cell polarity. Atg14 depletion results in autophagy defects with no effect on endocytosis or cell polarity, while the silencing of UVRAG phenocopies all but the autophagy defect of Atg6 depleted cells. Thus, our results indicate that the UVRAG-containing PI3K (III) complex is required for receptor downregulation through endolysosomal degradation and for the establishment of proper cell polarity in the developing wing, while the Atg14-containing complex is involved in autophagosome formation.

## 1. Introduction

Autophagy mediates the degradation of cytoplasm and organelles in eukaryotic cells. A set of evolutionarily conserved Atg proteins is required for autophagosome formation in yeast,* Drosophila*, and mammals [1, 2]. Autophagosomes then fuse with lysosomes to deliver their cargo for degradation, which requires the autophagosomal SNARE syntaxin 17 [3–5]. Activation of the Atg1 kinase complex leads to the initiation of autophagy, which is followed by the action of a class III phosphoinositide 3-kinase (PI3K) complex [6]. This lipid kinase complex is involved in multiple vesicle trafficking processes in yeast in addition to autophagy, namely, endosome maturation and biosynthetic transport to the vacuole, the yeast equivalent of lysosomes [7].

Autophagy related gene 6 (Atg6/Vps30 in yeast, Beclin 1 in mammals) is a core component of the Vps34 complex and is best known for its crucial role in the induction of autophagy [8, 9]. In addition to Atg6, the PI3K (III) core complex is composed of a regulatory subunit (vacuolar protein sorting 15—Vps15) and a catalytic subunit (Vps34) responsible for the production of phosphatidylinositol-3-phosphate (PI3P) from phosphatidylinositol (PI) [7, 10–12]. This membrane lipid localizes to early endosomes and the internal vesicles of multivesicular bodies (MVBs) in mammalian cells [13]. Vps34 and thus PI3P are required for the sorting of hydrolytic enzymes to the lysosome/vacuole [14], autophagosome formation, endocytic trafficking, and the regulation of cell polarity [15–18].

The core PI3K (III) complex is able to bind multiple regulator proteins and forms distinct complexes in yeast and mammalian cell lines. An Atg14-containing complex I is proposed to function in autophagy, whereas the UVRAG- (ultraviolet radiation resistance associated-) containing complex II is considered to be involved in endocytosis and vacuolar protein sorting [7, 10–12]. In yeast and mammalian cells, Atg14 is considered to be autophagy specific and is required for autophagosome formation [19–22], whilst UVRAG (also known as Vps38) has been shown to regulate late stages of autophagy and endocytic trafficking, and it may also interact with the class C Vps complex to promote vesicle tethering [12, 23–25]. However, Atg14 has recently been suggested to promote endocytic traffic based on shRNA experiments in cultured human cells [26].

*Beclin 1*, the mammalian homolog of yeast* ATG6*, is a potential haploinsufficient tumor suppressor gene, but such a role is not entirely evident based on data from human cancer patient samples [27–29].

Although the role of Atg6/Beclin 1 in autophagy is undoubted, its role in other processes, such as endocytosis, is less clear. It was shown that the expression of human Beclin 1 is able to rescue the autophagy, but not the vacuolar protein sorting defects in ATG6/VPS30 null mutant yeast [27]. This is further supported by the observation that in mammalian cells the maturation of cathepsin D in the lysosome is normal in cells that express little Beclin 1 [30]. This finding was supported by another group showing that the silencing of Beclin 1 suppresses autophagy, but not other PI3K (III) dependent processes [31]. This raises the question whether Beclin 1 is essential only for autophagy and dispensable for the role played by PI3K (III) in endocytic trafficking and lysosomal sorting [8]. In contrast, another work carried out on cultured human cells suggested that Atg6 is essential for endocytic degradation of epidermal growth factor receptor (EGFR) as a component of an UVRAG-containing PI3K (III) complex [32]. Although numerous key binding partners for Beclin 1 are involved in a variety of cell biological processes, such as endocytosis, endosomal sorting, and maturation, the direct evidence for the participation of Beclin 1 in these processes remains to be demonstrated* in vivo* [7].* Drosophila* Atg6 was shown to be required for autophagy [33, 34]. Furthermore, fluid-phase endocytosis is interrupted in Atg6 mutant larval fat body cells, and the number of Rab5 positive early endosomes is markedly decreased [34]. This latter finding is in contrast with observations in pupal wing cells where the RNAi of Vps15 resulted in the accumulation of late endosomes [17]. These findings raise the possibility that the roles of Atg6 may differ in distinct cell types even within one organism and that multiple PI3K (III) complexes may exist in* Drosophila*.

In this paper we show that, in* Drosophila*, Atg6 is an essential regulator of endolysosomal maturation in the developing wing. We show that endocytosis is seriously impaired in the developing Atg6 depleted pupal wing cells: dense multivesicular bodies and multilamellar bodies accumulate, which represent aberrant late endosomes and endolysosomes. We show that Atg6 as an endocytosis regulator is essential for the downregulation of multiple signaling pathways regulating wing development such as Notch and Wingless. The knockdown of Atg6 results in several serious defects in the development of the wing tissue which is a consequence of disorganization of cell adhesion proteins and disturbed cell polarity. In addition, we show that RNAi knockdown of UVRAG, but not of Atg14, phenocopies the endolysosomal trafficking and cell polarity defects seen in Atg6 loss-of-function cells. In the case of autophagy, we find that both Atg14 and Atg6 are required, whereas UVRAG appears to be largely dispensable.

## 2. Materials and Methods

### 2.1. *Drosophila* Strains and Genetics

Fly stocks used in this study are listed in Table S1 in the Supplementary Material available online at http://dx.doi.org/10.1155/2014/851349. Flies were raised on standard yeast/cornmeal/agar media, at 25°C, 50% humidity, and a 12-hour light/12-hour dark daily cycle, under uncrowded condition. To analyze the function of Atg6, UVRAG, and Atg14, transgenic RNAi flies were crossed to Bx^MS1094^Gal4; UAS-Dicer2 lines. Progeny was used for the examination of the morphology of the adult wings and for electron microscopy. For the generation of Atg6 mutant wings, in which the large portion of the wing tissue is derived from homozygous Atg6 null-mutant clone cells, we used a modified method of Newsome and colleagues [35]. Briefly, we used Bx^MS1094^Gal4 to drive the ubiquitous expression of FLP exclusively in wing disc cells, as we assumed that this system would provide continuous high levels of FLP activity throughout the proliferative phase of wing development, resulting in a high frequency of mosaicism. To increase the size of the clones, we used an FRT chromosome, where a Minute mutation was recombined onto the GFP marked FRT chromosome arm. As Minute mutations prevent the proliferation or survival of homozygous cells and retard the proliferation of heterozygous cells [36], we anticipated that wing tissue would mainly consist of Atg6 null-mutant cells. Therefore these wing discs or wings are referred to in the text as mutant discs or wings. In most cases, for fluorescent microscopy we used engrailed driven Gal4 (enGal4) to restrict the expression of the dsRNA-s to the posterior compartment of the developing wing, and since the area of the RNAi was marked by the expression of a fluorescent protein (GFP or RFP), the anterior part of the wing served as control. For null mutant clone generation P{neoFRT}82BAtg6^1^/TM6Tb flies were crossed to hs-FLP; P{neoFRT}82B, P{w+ Ubi-GFP(S65T)nls}3R/TM6Tb flies. Progeny was heat shocked for 2 hours at 37°C at the second larval stage. Flies were then kept at 25°C.

### 2.2. Antibodies

Antibodies used in this study are listed in Table S2 with the corresponding dilutions, applications, and references.

### 2.3. Histology and Microscopy

32 hour (after pupal formation (APF)) staged pupae were dissected and fixed with 4.0 (w/v)% paraformaldehyde (PFA) in PBS for 90 minutes at room temperature (RT), then were redissected in PBS to remove the wing cuticles. Third instar larval wing discs were dissected in ice cold PBS, then fixed with 4.0% PFA in PBS (60 min, RT). Pupal wings and wing discs were processed for immunofluorescence microscopy under the same conditions as follows: samples were incubated in 0.1% (v/v) Triton X-100 in PBS (PBTX, 30 min, RT), then in blocking solution (5.0% (v/v) FCS in PBTX). Samples were then incubated in the blocking solution completed with primary antibodies (overnight (ON), 4°C). Samples were then rinsed (3×), washed in PBTX (3 × 10 min, RT), and incubated in blocking solution (30 min, RT). Samples were then incubated with the corresponding secondary antibodies diluted in blocking solution. Washing steps were repeated, nuclei were stained with Hoechst 33342 (10 *μ*g/mL in PBS), and the samples were mounted in Vectashield (Vector). For rhodamine-phalloidin staining wings were fixed as described above, then were incubated in PBTX (10 min, RT). Wings were then incubated in blocking solution (1% FCS in PBTX), followed by rhodamine-phalloidin (0.5 *μ*g/mL) in blocking solution completed with Hoechst (40 min, RT). Wings were then washed extensively with PBS, then examined. For* ex vivo* endocytic trafficking assay, wing imaginal discs were dissected in ice cold M3 medium, then incubated with anti-Notch extracellular domain antibody (in M3, 4°C, 10 min), chased for 3 hours (in M3, RT), then washed extensively in PBS, and fixed as described above. Incorporated antibody was detected with the corresponding secondary antibody as described above. All reagents used for light microscopy were obtained from Sigma-Aldrich, otherwise indicated. TUNEL-assays were performed as in [37]. To capture images, we used a Zeiss Axioimager Z1 microscope equipped with an ApoTome unit using AxioCam MRm camera with AxioVision 4.82 software. GFP intensity profiles were created using Image J software. Primary images were edited using Adobe Photoshop CS5 software: area of interest was cropped, and if it was necessary, brightness and contrast were adjusted.

### 2.4. Ultrastructural Analysis

#### 2.4.1. Transmission Electron Microscopy

Thirty-hour (APF) staged pupae were dissected and fixed with 2% formaldehyde, 0.5% glutaraldehyde, 3 mM CaCl_2_, and 1% sucrose in 0.1 M Na-cacodylate, pH 7.4 (overnight, 4°C). Samples were then redissected in Na-cacodylate to remove the wing cuticles, and then the dissected wings were postfixed in 0.5% osmium tetroxide (60 min, RT) and in half-saturated aqueous uranyl acetate (30 min, RT), dehydrated in graded series of ethanol, embedded in LR white according to the manufacturer's instructions, and cured for 24 hours at 60°C. Ultrathin sections were stained with 4% uranyl acetate in 50% methanol (for 8 min) and lead citrate (for 3 min). Grids were analyzed in JEOL JEM 1011 transmission electron microscope operating at 60 kV. Images were taken using Olympus Morada 11 megapixel camera and iTEM software (Olympus). All reagents and materials used for electron microscopy were obtained from Sigma-Aldrich.

#### 2.4.2. Acid Phosphatase Cytochemistry

Pupal wings were fixed and dissected as described above, were washed in 0.05 M Na-acetate buffer pH 5.0 (3 × 5 min, RT), and then were incubated in Gömöri's medium (5 mM *β*-glycerophosphate and 4 mM lead nitrate dissolved in 0.05 M acetate buffer) for 30 min at RT. Samples were then washed in acetate buffer (3 × 5 min) and processed for electron microscopy as described above. Ultrathin sections were analyzed unstained. Substrate free medium was used for control experiments.

#### 2.4.3. DAB Staining

Pupal wings were fixed and dissected as described above and were washed in quenching buffer (30 min, 50 mM glycine and 50 mM NH_4_Cl in 0.05 M Tris buffer (TB), pH = 7.6), followed by a 10 min wash in TB. Wings then were preincubated in 1% H_2_O_2_ in TB in order to block endogenous peroxidases. Next, wings were washed extensively (3 × 10 min in TB) and then were preincubated with DAB (0.5 mg/mL DAB in TB, 10 min). Wings were then incubated in DAB reaction buffer (0.01% H_2_O_2_ and 0.5 mg/mL DAB in TB, 20 min), and DAB reaction was terminated by extensive washing (3 × 10 min in TB). Wings were then processed for electron microscopy as described above. Ultrathin sections were analyzed unstained. H_2_O_2_-free DAB reaction buffer was used for control experiments.

#### 2.4.4. Immuno-EM

Pupae were dissected and fixed with 4% formaldehyde, 0.05% glutaraldehyde, and 0.2% picric-acid in phosphate buffer (0.1 M PB, pH 7.4 overnight, and 4°C). Samples were then redissected in PB to remove the wing cuticles and were washed extensively with PB and free aldehyde groups were quenched with 50 mM glycine and 50 mM NH_4_Cl in PB. Wings were then postfixed in 1% uranyl acetate in 0.05 M maleate buffer (3 h, RT). Wings were then dehydrated in graded series of ethanol as follows: 25% EtOH (10 min, 0°C), 50% EtOH (10 min, 0°C), 70% EtOH (10 min, −20°C), 96% EtOH (20 min, −20°C), and Abs EtOH (2 × 60 min, −20°C). Samples were then infiltrated with pure LR white completed with 2% benzoyl peroxide as catalyst (24 hours, −20°C). Curing was performed with a homemade UV chamber using two 2 × 6 W UV lamp for 48 h (−20°C). Ultrathin sections (80–90 nm) were collected on formvar coated 100 mesh nickel grids. All the immunoreactions were carried out on humidified Parafilm coated 96 well plates on RT, otherwise indicated. The following procedure was performed: (1) 5% H_2_O_2_ for 1 min; (2) biDW (bidistilled water) for 3 × 5 min; (3) 0.1% NaBH_4_ in TBS (pH: 7.6) for 10 min; (4) 50 mM glycine in TBS for 30 min; (5) TBS for 3 × 5 min; (6) 10% FCS in TBS for 30 min; (7) anti-GFP in 5% FCS-TBS overnight at 4°C; (8) 2% FCS in TBS for 3 × 5 min; (9) 18 nm gold-conjugated secondary antibody in 2% FCS in TBS for 90 min; (10) 3 × 5 min TBS; (11) 1% glutaraldehyde in TBS for 10 min; (12) extensive wash with biDW. Ultrathin sections were stained with uranyl acetate (for 15 min) and lead citrate (for 1 min).

### 2.5. Quantification and Statistical Analysis

From images of pupal wings we randomly took sample quadrates of 100 × 100 pixels using Adobe Photoshop CS5 extended v.12.0 software from both control and Atg6-, UVRAG-, or Atg14-depleted domains of the wings. Percentage of the area covered by the fluorescent markers indicated above was measured using Image J software. In case of wing discs, we used sample areas of 75 × 75 pixels from the wing pouch exclusively. Percent of area covered by the signal of anti-Delta or anti-Notch antibodies was measured with the method described formerly. Effect of Atg6 null mutations on Notch EC domain localization was quantified by selecting 30 × 30 pixels measurement areas containing clone or control cells exclusively. In sampling, we chose the nearest neighboring area (in less than a distance of 100 pixels) of the selected clone cell group. The percentage of signal-covered area was measured using Image J. We performed the statistical analysis applying the indicated tests and constituted the box plot figures using IBM SPSS Statistics 21 software. On box plots, bars show the data ranging between the upper and lower quartiles; median is indicated as a horizontal black line within the box. Whiskers plot the smallest and largest observations, while dots and asterisks indicate outliers. *P* < 0.05 was considered to be significant. NS means *P* > 0.05, ∗ means *P* < 0.05, ∗∗ means *P* < 0.01, and ∗∗ means *P* < 0.001. For details and results of statistical analyses, see Table S3.

### 2.6. RT-PCR

RT-PCR experiments were performed following standard protocols. Total RNA and cDNA were prepared using Direct-zol RNA MiniPrep (R2051-Zymo Research) and RevertAid First Strand cDNA Synthesis Kit (K1621, Thermo Scientific) from RNAi and control L3 larvae, and these were used as template for PCR reactions with the following primers: Atg6: 5′-CGGAGTTATCTTTGCCCATCTA-3′ and 5′-GGCGTTGATCTCTGACCAGT-3′, UVRAG: 5′-CCACACTGGTGTTGGAGCTA-3′ and 5′-CCGAACGGCAAATGCGTTGA-3′, and Atg14: 5′-CTGGGTCTTCTGGACAGCAT-3′ and 5′-GAGTTTTCGTCCTCTGACTC-3′. Actin was used as loading control (5′-GTCGCTTAGCTCAGCCTCG-3′ and 5′-TAACCCTCGTAGATGGGCAC-3′).

## 3. Results

### 3.1. *Drosophila* Atg6 Is Required for PI3P Production, Endosomal Trafficking, and Lysosome Maturation, Similar to UVRAG

**Figure 1:**
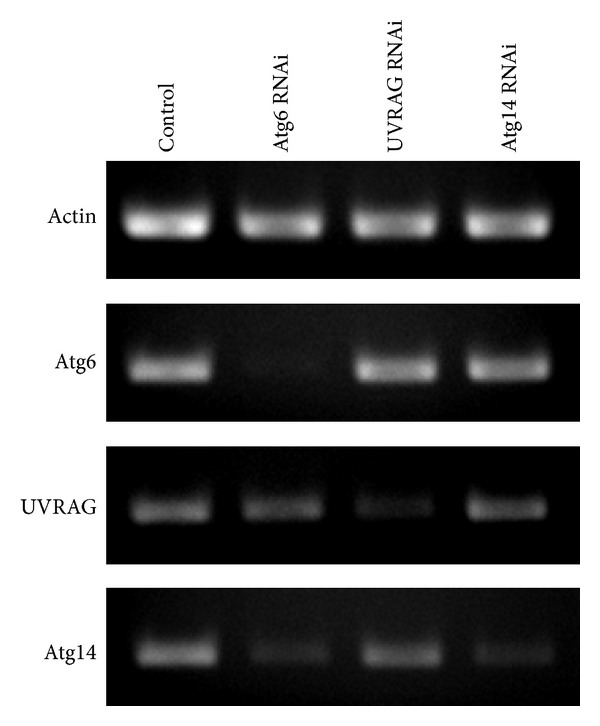
RT-PCR analysis of Atg6, UVRAG, or Atg14 transcripts from animals expressing transgenic RNAi constructs. Expression of Atg6, UVRAG, or Atg14 RNAi by tubGal4 strongly reduced the mRNA level of the corresponding genes. Interestingly, we found that the RNAi of Atg6 also decreased the mRNA level of Atg14.

To analyze the functional role of Atg6, UVRAG, and Atg14 in* Drosophila*, we first established that we can selectively inhibit the expression of these genes by transgenic RNAi. RT-PCR experiments revealed that systemic expression of Atg6, UVRAG, or Atg14 dsRNA strongly reduced the mRNA level of the corresponding genes. Interestingly, we found that the depletion of Atg6 also reduced the mRNA level of Atg14 (Figure 1), although not to the extent seen in the case of Atg14 RNAi. While the reason for this observation is not known, it is in line with a previous report showing that Beclin 1 siRNA treatment reduces Atg14 expression levels in cultured cells [32].

#### 3.1.1. Atg6 and UVRAG Are Required for PI3P Production

**Figure 2:**
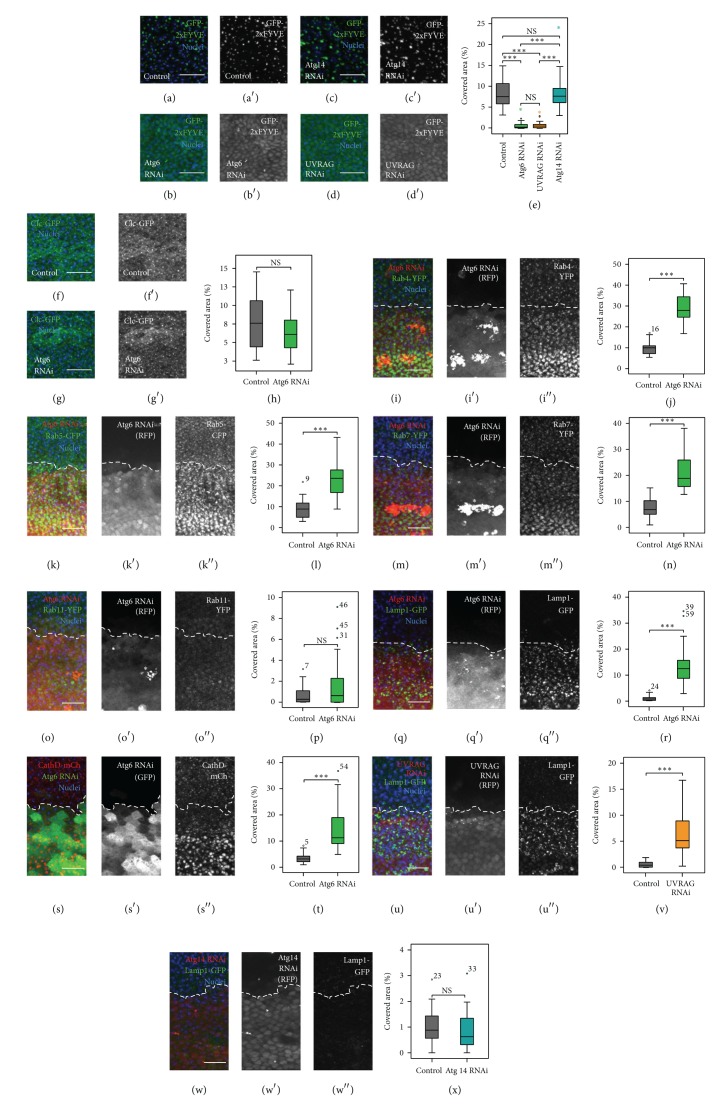
Knockdown of Atg6 and UVRAG. (a) The PI3P marker GFP-2xFYVE localized to dots and vesicle-like structures in wild type pupal wings. (b), (d) In Atg6 or UVRAG depleted wings the GFP-2xFYVE became dispersed within the cytoplasm, indicating a failure in PI3P production. (c) In contrast to Atg6 or UVRAG RNAi wings the localization of GFP-2xFYVE did not differ from controls in Atg14 RNAi wings. (e) Quantification of (a)–(d). (f), (g) In control wings clathrin coated vesicle marker Clc-GFP showed punctate pattern which was unaffected by Atg6 RNAi. (h) Quantification of (f), (g). (i)–(w) Images from pupal wings expressing endosomal and lysosomal fluorescent markers controlled by a constitutive promoter (tubulin promoter for Rab-FP-s and Lamp1-GFP, cathD promoter for CathD-mCherry) and expressing RNAi constructs by enGal4. The region of RNAi is marked by the coexpression of RFP or GFP and the borderline of the region of RNAi is indicated with a dashed white line. (i)–(p) The number of structures positive for endosomal markers ((i): Rab4-YFP; (k): Rab5-CFP; (m): Rab7-YFP, except (o): Rab11-YFP) was significantly increased in Atg6 RNAi cells. (j), (l), (n), and (p) Quantification of (i), (k), (m), and (o), respectively. (q)–(t) The number of lysosomes marked by Lamp1-GFP or CathD-mCherry was significantly increased in Atg6 depleted cells. (r), (t) Quantification of (q), (s), respectively. (u) UVRAG RNAi resulted in the accumulation of Lamp1-GFP structures very similar to Atg6 RNAi. (v) Quantification of (u). (w) RNAi of Atg14 did not alter the number of Lamp1-GFP positive dots. (x) Quantification of (w). On images from (i) to (w), the intensity of the markers is enhanced by immunostaining the wings with an anti-GFP or anti-mCherry. Note the presence of RFP positive hemocytes under the epithelia on (i), (m), and (o) (marked by asterisks). On box plots, bars show the data ranging between the upper and lower quartiles; median of the signal covered areas is indicated as a horizontal black line within the box. Whiskers plot the smallest and largest observations, while dots and asterisks indicate outliers. NS means *P* > 0.05 and ∗∗∗ means *P* < 0.001. For details and exact *P* values of statistical analyses, see Table S3. For genotypes see Table S4. Scale bars represent 15 *μ*m.

As PI3K (III) core components Vps15 and Vps34 have been shown to promote endocytosis in* Drosophila* [15, 17] and Vps34 has been shown to physically interact with Atg6 [15], first we examined the* in vivo* activity of the PI3K (III) complex in Atg6 knockdown cells. For this purpose we used animals in which two FYVE domains fused to GFP (GFP-2xFYVE) was expressed. This reporter protein selectively binds to PI3P containing membranes in wild type cells (Figure 2(a)). We found that in Atg6 RNAi pupal wing cells the GFP-2xFYVE loses its localization to endosomes but rather becomes dispersed within the cytoplasm. This suggests that PI3K (III) activity is dramatically reduced in pupal wing cells when cells lack Atg6, since in the absence of PI3P this marker is no longer able to associate with endosomal membranes (Figures 2(b) and 2(e)). This is in line with the results obtained by Shravage and colleagues, as Atg6 depleted larval fat body cells showed a very similar phenotype [34]. We found that in contrast to Atg6, Atg14 may not be required for PI3P production in pupal wing cells as Atg14 RNAi had no significant effect on GFP-2xFYVE localization, whilst UVRAG RNAi had very similar effect to Atg6 RNAi (Figures 2(c), 2(d), and 2(e)). Thus, PI3P is likely associated with endosomes rather than autophagosomes in this tissue.

#### 3.1.2. Depletion of Atg6 and UVRAG Results in the Accumulation of Defective Endolysosomes

While there are some controversial data that Atg6 is required for endocytosis, detailed examination of the effect of Atg6 loss-of-function on different endosome populations is lacking. Therefore we next investigated the effects of Atg6 RNAi on several endosomal and lysosomal markers in pupal wings. First we used the endocytosis marker clathrin light chain-GFP (Clc-GFP) which labels clathrin-coated vesicles, and we could not detect any difference between Atg6 RNAi cells and control cells (Figures 2(f)–2(h)). Interestingly, that was not the case when later stages of endocytosis were examined. For this purpose we used animals expressing endosomal and lysosomal reporters controlled by a constitutive promoter and expressing RNAi constructs by enGal4. As the expression of the RNAi was restricted to the posterior compartment of the wing, the anterior part could serve as control. We found a significantly increased area of Rab4-YFP, Rab5-CFP, and Rab7-YFP positive dot-like structures, and mostly similar number of Rab11-YFP positive dots in the regions where the dsRNA of Atg6 was expressed. This implies that inactivation of PI3K (III) by Atg6 RNAi results in the accumulation of early and late endosomes (Figures 2(i)–2(p)). These results together suggest that Atg6 as a component of* Drosophila* PI3K (III) core complex is involved in endosomal maturation.

As late endosomes mature into lysosomes, their luminal pH continues to decrease and their membranes acquire lysosome specific proteins such as Lamp (Lysosome-associated membrane protein), and after fusion with lysosomes this process terminates as lysosomal hydrolases (such as cathepsins or acid phosphatases) degrade the luminal contents of these secondary lysosomes. Interestingly, it was found that depletion of Atg6 in pupal wing cells results in the massive accumulation of Lamp1-GFP positive and cathepsin D positive granules (Figures 2(q)–2(t)). A similar phenomenon was observed when the effect of the UVRAG RNAi was examined as cells lacking UVRAG accumulated numerous Lamp1-GFP positive granules (Figures 2(u) and 2(v)). In contrast, Atg14 RNAi cells had the same phenotype as control cells (Figures 2(w) and 2(x)). These results indicate that the UVRAG-containing lipid kinase complex is involved in endolysosomal maturation.

#### 3.1.3. Electron Microscopy of Cells Lacking Atg6 or UVRAG Reveals the Accumulation of Abnormal Endolysosomes

**Figure 3:**
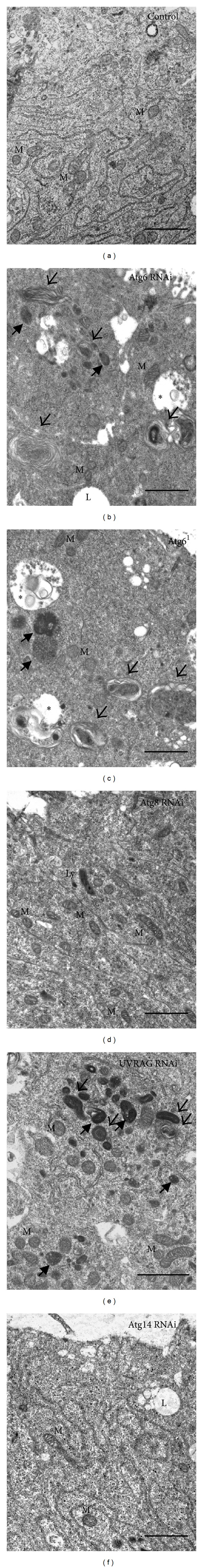
Electron microscopy of pupal wings. (a) Ultrastructure of control cells. (b) Depletion of Atg6 by RNAi resulted in the massive accumulation of enlarged multivesicular bodies (asterisks), dense multivesicular-body like structures (arrows), and multilamellar bodies (open arrows). (c) All of these structures could be found in Atg6 mutant pupal wings. (d) The depletion of Atg8a did not alter the ultrastructure of pupal wing cells. (e) Similar to Atg6, UVRAG RNAi also resulted in the accumulation of aberrant endolysosome-like structures (MVBs: arrows, MLBs: open arrows). (f) The depletion of Atg14 did not alter the ultrastructure of pupal wing cells. M: mitochondria, L: lipid droplet, and Ly: lysosome. For genotypes, see Table S4. Scale bars represent 1 *μ*m.

Electron microscopy revealed that many aberrant late endosome-like structures, such as enlarged lucent or dense multivesicular body- (MVB-) like structures and multilamellar bodies (MLB) accumulated the apical cytoplasm of Atg6 RNAi pupal wing cells, whereas these structures were completely absent in control cells (Figures 3(a) and 3(b)). The ultrastructure of Atg6 null mutant cells was similar to Atg6 RNAi cells, further confirming this observation (Figure 3(c)). In order to decide whether this phenotype originates from the lack of autophagy, the RNAi of Atg8a was also examined. We found that the ultrastructure of the Atg8a RNAi cells was very similar to wild type cells (Figure 3(d)), suggesting that the abnormal structures in Atg6 depleted cells were not derived from the lack of autophagy. As it was expected from the Lamp1-GFP phenotype, the UVRAG RNAi pupal wing cells also accumulated numerous aberrant endolysosome-like structures, similar to Atg6 loss-of-function cells (Figure 3(e)). In contrast the ultrastructure of Atg14 RNAi cells was completely indistinguishable from wild type or Atg8a RNAi cells (Figure 3(f)).

**Figure 4:**
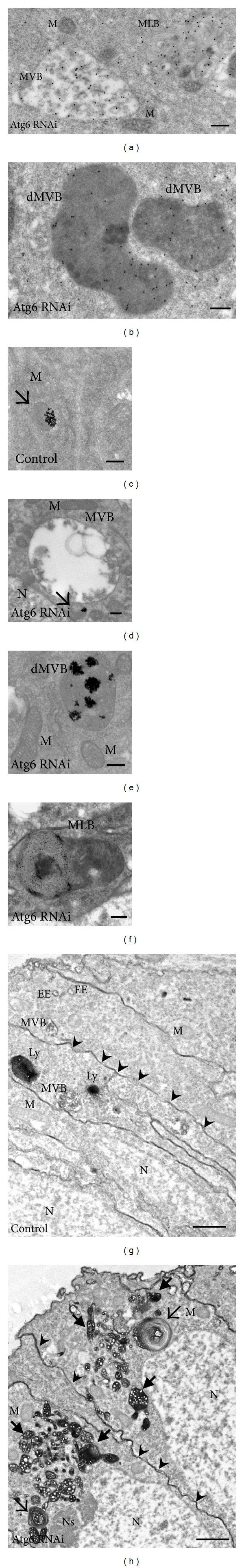
Identification of the abnormal organelles of Atg6 RNAi cells. (a), (b) Immunogold labeling of Atg6 RNAi cells with anti-GFP antibody to detect Lamp1-GFP. MVBs, dMVBs, and MLBs were positive to Lamp1-GFP. (c) Enzymecitochemistry revealed small, dense acid phosphatase positive structures (open arrow) in wild type pupal wing cells. Based on the size and morphology, these structures were considered as primary lysosomes. All other organelles were devoid of reaction product. (d) Electron-lucent MVBs in Atg6 depleted cells never contained acid phosphatase reaction product but were often found in the vicinity of acid phosphatase positive primary lysosomes (open arrow). (e) A dense multivesicular body-like structure (dMVB) showing acid phosphatase reaction product in an Atg6 RNAi pupal wing cell. (f) A multilamellar body (MLB) in Atg6 depleted cell positive to acid phosphatase. (g), (h) DAB-staining of pupal wings expressing plasma membrane localized horseradish peroxidase enzyme (HRP-CD2). (g) In control cells plasma membrane showed a prominent DAB staining (arrowheads), just as organelles with plasma membrane origin, such as early endosomes (EE), multivesicular bodies (MVB), and secondary lysosomes (Ly) as well. (h) The accumulated organelles in Atg6 depleted cells: the multivesicular bodies (arrows) and multilamellar bodies (open arrows) were all positive to DAB. M: mitochondria, MVB: multivesicular body, dMVB: dense multivesicular body-like structure, MLB: multilamellar body, N: nucleus, Ns: nucleolus, and Ly: lysosome. For genotypes, see Table S4. Scale bars represent 200 nm in (a)–(f) and 500 nm in (g), (h).

Postembedding immunocytochemistry showed that the aberrant late endosomes and endolysosomes in Atg6 depleted cells were positive for LAMP1-GFP, indicating that they could be immature or malfunctioning lysosomes (Figures 4(a) and 4(b)). Interestingly, many of the MVBs were often found in the close vicinity of small dense vesicles. These small structures always showed acid phosphatase activity and were also found in wild type cells; therefore they likely represent primary lysosomes (Figures 4(c) and 4(d)). Furthermore, enzyme cytochemistry also revealed that a notable portion of the dense MVBs (58%, *N* = 85) and all of the MLBs showed acid phosphatase activity as well, whilst the electron-lucent MVBs never did (Figures 4(d)–4(f)). This raised the possibility that the sorting of lysosomal enzymes into MVBs and/or the maturation dynamics of MVBs into secondary lysosomes were seriously compromised in Atg6 depleted cells. As disrupted primary lysosomes or Golgi vesicles may also show acid phosphatase activity, a plasma membrane localized horseradish peroxidase enzyme (HRP-CD2) was expressed in the developing wing, to confirm the endosomal origin of the aberrant lysosome-like compartments in Atg6 depleted cells. This reporter protein can be visualized with routine diaminobenzidine (DAB) staining and due to the stability of the protein, this method allows one to label all membranes with plasma membrane origin, including all kinds of endosomes and secondary lysosomes as well [38]. We found that the aberrant structures accumulated in the Atg6 RNAi cells were all positive to DAB; therefore their endosomal origin is clearly confirmed (Figures 4(g) and 4(h)). Taken together our results strongly suggest that Atg6 has essential functions in endosomal and lysosomal maturation and sorting.

### 3.2. Atg6 and Atg14 Are Required for Autophagy in the Wing, Unlike UVRAG

**Figure 5:**
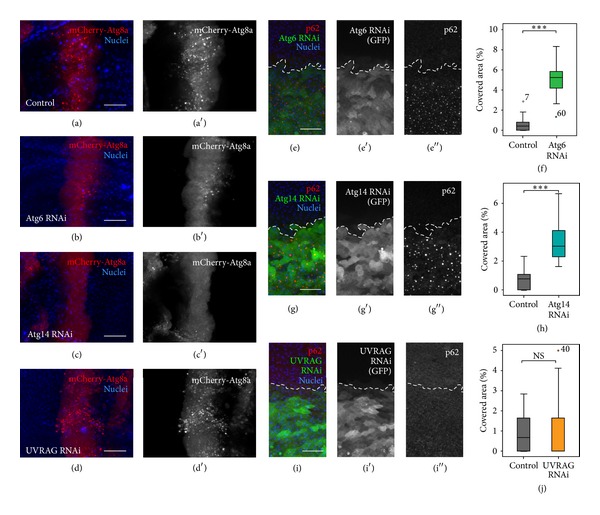
Atg6 and Atg14 are required for autophagy in contrast to UVRAG. (a)–(d) Larval wing discs expressing RNAi with transgenic Myc along with the autophagy marker mCherry-Atg8 in the patched (ptc) domain using ptcGal4. (a) In controls the expression of Myc induces massive autophagy in the ptc domain. (b) Atg6 or (c) Atg14 RNAi inhibited Myc-induced autophagy as the pattern of mCherry-Atg8 became dispersed in the citoplasm, whereas (d) UVRAG RNAi did not alter the pattern of the autophagy marker. (e), (g), and (i) Pupal wings expressing RNAi constructs by enGal4, immunostained against the selective autophagic cargo protein p62. (e) Atg6 or (g) Atg14 RNAi resulted in the massive accumulation of p62 aggregates. (i) UVRAG RNAi cells did not accumulate p62 aggregates. (f), (h), and (j) Quantification of (e), (g), and (i), respectively. On box plots, bars show the data ranging between the upper and lower quartiles; median of the signal covered areas is indicated as a horizontal black line within the box. Whiskers plot the smallest and largest observations, while dots and asterisks indicate outliers. NS means *P* > 0.05 and ∗∗∗ means *P* < 0.001. For details and exact *P* values of statistical analyses, see Table S3. For genotypes, see Table S4. Scale bars represent 25 *μ*m in (a)–(d) and 15 *μ*m in (e), (g), and (i).

As Atg6 and Atg14 have been shown to be required for autophagy in the larval fat body [34, 39], next we examined the effect of Atg6, Atg14, and UVRAG depletion on autophagy in the wing imaginal disc and pupal wing.* Drosophila* Myc has been shown to efficiently induce autophagy in various* Drosophila* tissues [40]. We overexpressed Myc in the patched (ptc) domain of the wing disc along with the autophagy marker mCherry-Atg8a, using ptcGal4. We found that compared to controls (Figure 5(a)), RNAi of Atg6 or Atg14 inhibited Myc-induced autophagy (Figures 5(b) and 5(c)), whilst UVRAG RNAi wing discs showed a phenotype similar to controls (Figure 5(d)). The role of Atg6 and Atg14 but not UVRAG in autophagy was confirmed by immunostaining pupal wings against the selective autophagy cargo p62/Ref(2)P. This protein can be used to detect autophagy defects, as in such cases cells accumulate p62 aggregates [41–43]. We found a significantly increased number of p62 positive aggregates in the regions where the dsRNA of Atg6 (Figures 5(e) and 5(f)) or Atg14 (Figures 5(g) and 5(h)) was expressed. In contrast, UVRAG RNAi cells did not accumulate p62 (Figures 5(i) and 5(j)). These results together suggest that Atg6 and Atg14 are required for autophagy in wing discs and wings, while UVRAG is dispensable for autophagy in these tissues.

### 3.3. Atg6 Depletion Results in Defective Endocytic Degradation of Signaling Molecules and Enhances Notch Signaling, Similar to UVRAG

#### 3.3.1. Depletion of Atg6 or UVRAG Increases the Endosomal Retention of Notch, Unlike Atg14

**Figure 6:**
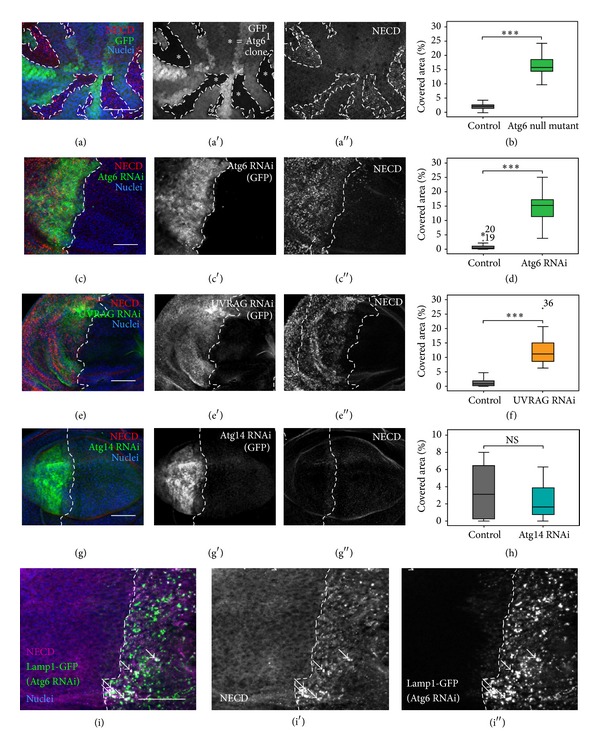
Impaired endocytic degradation of Notch in Atg6 depleted cells. (a) In 3rd instar larval wing discs Atg6 null mutant clones (marked by the absence of GFP, asterisks) accumulate the extracellular domain of Notch (NECD). (b) Quantification of (a). (c), (e), and (g) Imaginal wing discs expressing dsRNA (Atg6, Atg14) or siRNA (UVRAG) by enGal4 were immunostained against NECD. The region of RNAi is marked by the coexpression of GFP and the borderline of the region is indicated with a dashed white line. Immunostain revealed that NECD accumulated in small, numerous puncta in the case of (c) Atg6 RNAi and (e) UVRAG RNAi, (g) but not in Atg14 RNAi. (d), (f), and (h) Quantification of (c), (e), and (g), respectively. (i) Live trafficking assay for Notch in cultured wing imaginal discs. Atg6 RNAi region is marked by the coexpression of the lysosome marker Lamp1-GFP. In controls, surface-bound Notch was internalized then degraded after 3 h. In contrast, in Atg6 depleted cells Notch was internalized normally but trapped in vesicular structures even at 3 h of chasing. Arrows indicate Notch signal colocalizing with Lamp1-GFP. On box plots, bars show the data ranging between the upper and lower quartiles; median of the signal covered areas is indicated as a horizontal black line within the box. Whiskers plot the smallest and largest observations, while dots and asterisks indicate outliers. NS means *P* > 0.05 and ∗∗∗ means *P* < 0.001. For details and exact *P* values of statistical analyses, see Table S3. For genotypes, see Table S4. Scale bars represent 50 *μ*m.

As it has been shown that Notch signaling is enhanced in mutants that increase endosomal retention [44], we assumed that the accumulation of late endosomes/endolysosomes in Atg6 depleted cells could result in enhanced Notch signaling. To assess this possibility, first we examined the cellular localization of Notch and Delta (the ligand of Notch). As expected, Notch and Delta both accumulated in small, numerous puncta in the absence of Atg6, indicating that these molecules could be restrained from lysosomal degradation (Figures 6(a)–6(d), Figures S1(a) and S1(b)). Whilst UVRAG RNAi resulted in a similar phenotype to Atg6 RNAi, the depletion of Atg14 had no detectable effects on the localization of Notch (Figures 6(e)–6(h)). To further examine the effects of Atg6 RNAi, time-course* ex vivo *experiments were carried out. We incubated an antibody against the extracellular domain of Notch with live wing imaginal disc cells, and the surface bound antibody was allowed to get internalized and degraded. We found that the cell surface localized Notch was internalized normally but became trapped in vesicular structures in Atg6 depleted cells even at 3 h of chasing (Figure 6(i)). On the other hand, control cells successfully took up and degraded Notch over this time. Many of the Notch containing granules colocalized with the lysosome marker Lamp1-GFP in Atg6 RNAi cells, further suggesting that the degradation of Notch is impaired when cells lack Atg6 (Figure 6(i)).

#### 3.3.2. Notch Signaling Is Enhanced in Atg6 or UVRAG Loss-of-Function Cells, Unlike Atg14

**Figure 7:**
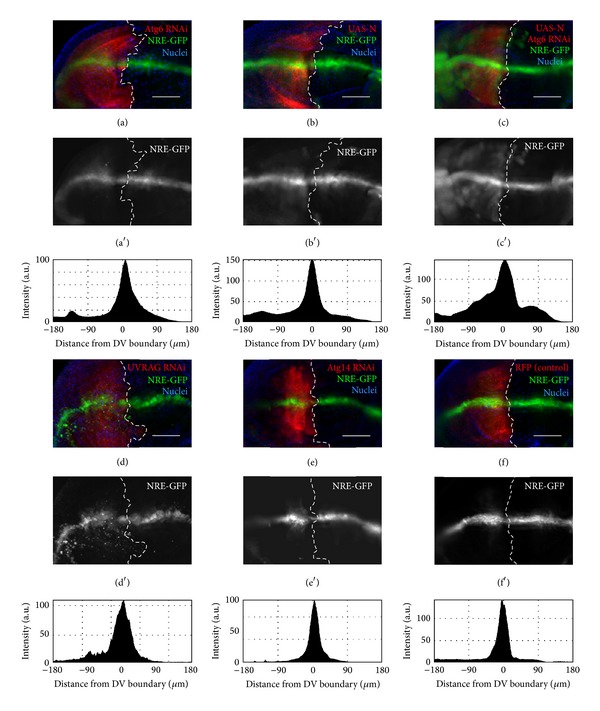
Enhanced Notch signaling in Atg6 RNAi and UVRAG RNAi cells. Larval wing discs expressing RNAi with or without transgenic Notch. UAS-myrRFP was used to mark enGal4-specific region. Notch response element- (NRE-) EGFP was used to show Notch transcriptional activity. (a) Atg6 RNAi and (b) Notch overexpression lead to enhanced Notch activity. (c) This effect was further increased as coexpression of Atg6 RNAi and Notch strongly enhanced the reporter expression. (d) UVRAG enhanced Notch transcriptional activity similarly to Atg6 RNAi (e) RNAi of Atg14 did not alter the reporter expression. (f) Control disc. Histograms show the intensity profiles of the NRE-GFP expression in the enGal4-specific regions. Note that the peaks are broader in panels (a)–(d), compared to panels (e), (f). For genotypes, see Table S4. Scale bars represent 50 *μ*m.

To examine Notch signaling activity in Atg6, UVRAG, or Atg14 loss-of-function cells, a Notch response element, EGFP (NRE-GFP), was used. The expression of this reporter depends on the transcriptional activity of Notch; therefore it can be used to show Notch signaling activity [45]. We found that compared to controls, the RNAi of Atg6 results in the enhancement of the reporter expression, which observation was very similar to the effect of UVRAG RNAi or wild type Notch protein overexpression (Figures 7(a), 7(b), 7(d), and 7(f)). The effect of Atg6 RNAi on the reporter expression was remarkably enhanced when wild type Notch protein was coexpressed in the wing imaginal disc cells (Figure 7(c)), further indicating that Notch signaling is highly activated in the lack of Atg6. In contrast to Atg6 or UVRAG, the RNAi of Atg14 had no significant effect on the reporter expression (Figure 7(e)).

#### 3.3.3. Depletion of Atg6 or UVRAG Increases the Endosomal Retention of Wingless, Unlike Atg14

**Figure 8:**
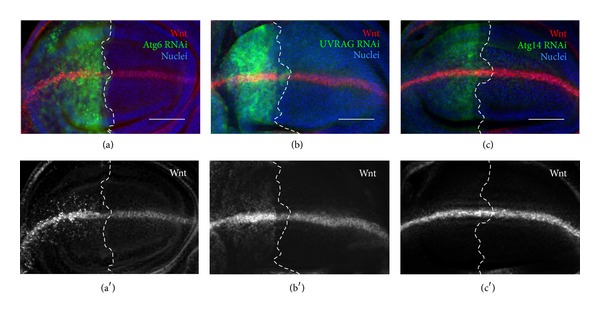
Impaired endocytic degradation of Wnt in Atg6 or UVRAG depleted cells. Imaginal wing discs expressing RNAi by enGal4 were immunostained against Wnt. The region of RNAi is marked by the coexpression of GFP and the borderline of the region is indicated with a dashed white line (a), (b) RNAi of Atg6 and UVRAG resulted in the accumulation of Wnt in small, numerous intracellular puncta. (c) RNAi of Atg14 did not affect Wnt localization. For genotypes, see Table S4. Scale bars represent 50 *μ*m.

As endocytosis is required for proper Wingless (Wnt) signaling as well [46–48], the localization of this protein was also examined. We found that similar to Notch, Wnt also accumulated in small puncta in Atg6 or UVRAG RNAi cells, while Atg14 RNAi had no detectable effect on the pattern of Wnt (Figure 8). This suggests that Atg6 and UVRAG may be required for regulating several other signaling pathways besides Notch, unlike the Atg14-containing PI3K (III) complex.

### 3.4. Depletion of Atg6 and UVRAG Causes Similar Malformations in the Developing Wing

**Figure 9:**
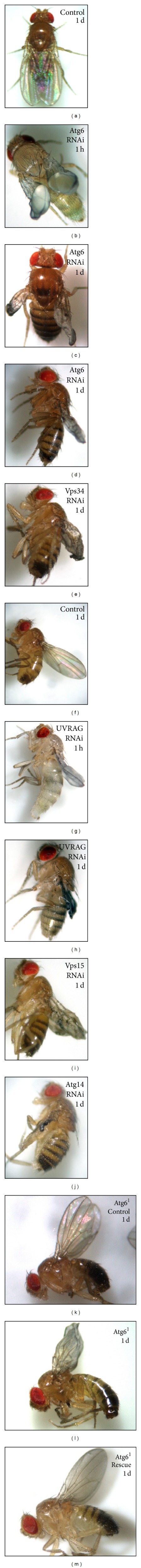
Atg6 and the other members of PI3K (III) are required for normal wing development: (a), (f) 1-day-old wild type flies. (b) Newly hatched Atg6 RNAi animals developed serious malformations, blisters in their wings. (c), (d) In 1-day-old Atg6 RNAi animals, the blisters had become collapsed; thus the wings were creased and heavily distorted. (g), (h) The RNAi of UVRAG completely mimiced the effect of Atg6 RNAi. (e), (f) Positive control animals: Vps34 RNAi and Vps15 RNAi flies, respectively. (j) Unlike the other members of PI3K (III) complex, the RNAi of Atg14 causes a vestigial-like morphology rather than blistering or creasing. (k) The Atg6 null mutant wings are heavily distorted and creased, (l) compared to the control wings lacking the mutation or (m) expressing an Atg6 transgene. 1 h: 1 hour; 1 d: 1 day after emerging from puparia, respectively. For genotypes, see Table S4.

As mutations in the endocytotic machinery can lead to the disturbance of cell polarity as well [17, 49] and these kinds of mutations commonly affect Notch signaling as well [44, 50], next we examined the overall wing morphology of RNAi or Atg6 null mutant animals. We found that compared to controls, the wing specific depletion of Atg6 and UVRAG by RNAi causes severe malformations of the tissue (Figures 9(a)–9(d), 9(f)–9(h), Figure S2), as the wing became blistered or heavily creased. This effect was very similar to Vps15 and Vps34 RNAi (Figures 9(e) and 9(i)), which were previously shown to be required for wing development [17]. As the Atg6 null mutant animals die during the late third larval or early pupal stages [34], mitotic recombination technique was used to generate completely null mutant adult wings. We found that Atg6 null mutant wings also exhibited a heavily creased morphology, which effect could be rescued by the expression of an Atg6 transgene (Figures 9(k)–9(m)). In contrast, Atg14 RNAi caused a vestigial-like effect rather than blistering or creasing (Figure 9(j), Figure S2). As the malformations of the RNAi wings could be the consequence of increased cell death in the developing wing tissue, wing discs were stained against cleaved Caspase-3, and TUNEL assays were performed to detect apoptosis. We found that, in Atg14 RNAi discs, numerous cells underwent apoptosis (Figures S3(a), S3(b), S3(e), and S3(f)), which could explain the wing phenotype of Atg14 RNAi adults. In contrast, in Atg6 or UVRAG RNAi discs no cleaved Caspase-3 or TUNEL positive cells could be detected (Figures S3(c), S3(d), S3(g), and S3(h)).

**Figure 10:**
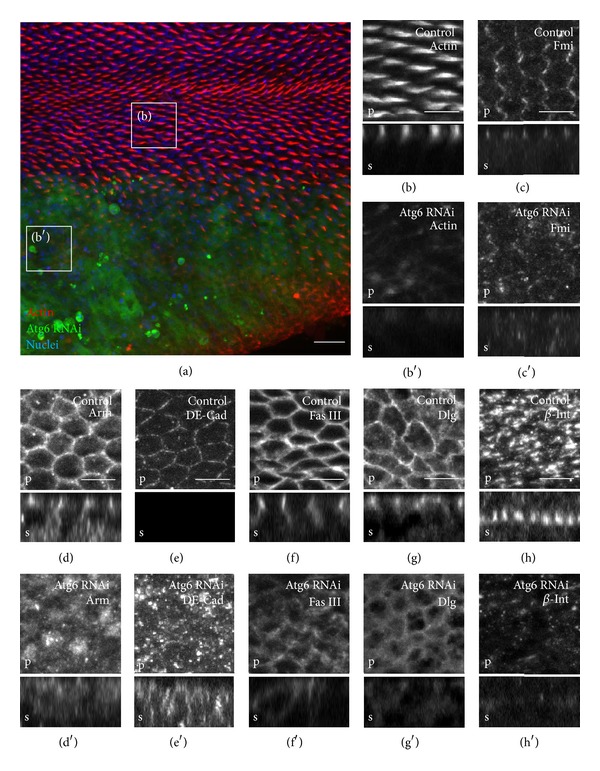
Polarity and membrane protein localization defects of Atg6-knockdown pupal wings. Semiconfocal immunodetection showing XY (marked by a letter p (plan view) at the bottom left corners) and XZ sections (marked by a letter s (side view) at the bottom left corners) in the plane of pupal wings at 32 h APF. Regions of controls are shown in boxes marked by a single character at the upper left side, whereas the regions where dsRNA for Atg6 was expressed by en-Gal4 driver are shown in boxes marked by a character at the upper left side with an apostrophe. (a) Low magnification image of a pupal wing with rhodamine-phalloidin stain showing the regions from where the high magnification images were taken. The expression of the dsRNA by enGal4 is restricted to the posterior compartment of the wing (marked by the coexpression of GFP), and anterior side serves as control. Small boxes surround areas of images (b) and (b′) in which rhodamine-phalloidin stain reveals that control wing hairs are well-developed and very regularly oriented towards the distal end of the wing. In contrast the wing hairs are poorly developed or completely absent in the regions of the RNAi. (c)–(e) In controls Fmi, Arm, and DE-Cad show a very pronounced zonula adherens (ZA) localization; furthermore Fmi shows a very regular planar cell polarity pattern as well, whereas all of them show intracellular punctuation and irregular localization in the Atg6 depleted wing regions. (f), (g) Septate junction proteins Fas III and Dlg show very typical lateral localizations as Dlg is mainly localized at the apical regions of septate junctions (SJ), whilst the domain of Fas III is expanded throughout the whole SJ. In contrast, in Atg6 RNAi cells the amounts of these proteins lowered and both show less pronounced SJ localization. (h) At this developmental stage the prospective wing intervein cells form large, *β*-integrin (*β*-int) containing basal junctions between the two epithelial layers of the wing, but this process is dramatically blocked when the level of Atg6 is reduced and wing cells seem to be unable to develop these structures. For genotype, see Table S4. Scale bars represent (a) 10 *μ*m and (b)–(h) 5 *μ*m.

Vps15, an adaptor subunit of PI3K (III), was shown to be required for the transport and sorting of several membrane proteins to the appropriate cell adhesion structures [17]. This raised the possibility that Atg6 and UVRAG could function together with Vps15 in this setting as well. As the blistering and creasing observed in the adult wing experiments could be a result of disrupted epithelial polarity of the wing cells, immunofluorescence microscopy was performed on the developing pupal RNAi wings against several cell adhesion molecules. For this purpose we used engrailed (en) promoter driven Gal4 to restrict the expression of the RNAi constructs to the posterior compartment of the developing wing; therefore the anterior compartment served as control (Figure 10(a)).

#### 3.4.1. Atg6 and UVRAG Regulate the Localization of Zonula Adherens Proteins

**Figure 11:**
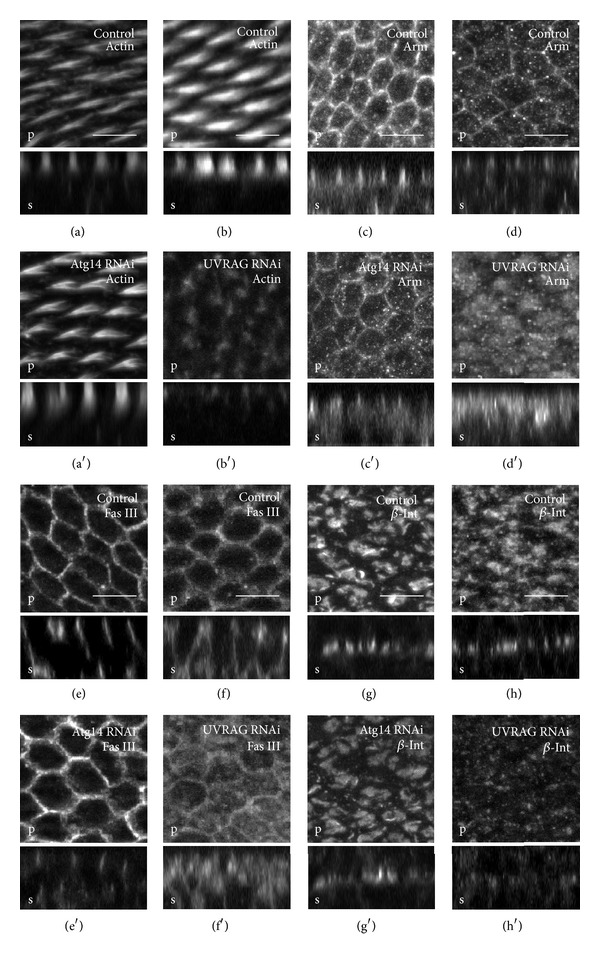
RNAi of UVRAG but Atg14 alters cell polarity and membrane protein localization. Semiconfocal immunodetection showing XY (marked by a letter p (plan view) at the bottom left corners) and XZ sections (marked by a letter s (side view) at the bottom left corners) in the plane of pupal wings at 32 h APF. Regions of controls are shown in boxes marked by a single character at the upper left side, whereas the regions where dsRNA for Atg6 was expressed by en-Gal4 driver are shown in boxes marked by a character at the upper left side with an apostrophe. (a), (c), (e), and (g) Immunodetection reveals no obvious differences of the pattern of wing hairs and the localization of Fas III, Arm, and *β*-int between control and Atg14 depleted regions of pupal wings. (b), (d), (f), and (h) In contrast to Atg14, the RNAi of UVRAG dramatically alters wing hair development and the localization of membrane proteins (Fas III, Arm, and *β*-int) and these phenotypes are very similar to those induced by Atg6 RNAi as was shown in Figure 2. For genotypes, see Table S4. Scale bars represent 5 *μ*m.

First, the zonula adherens (ZA) components were examined and the developing wings were immunostained against Flamingo (Fmi), DE-cadherin (DE-cad), and Armadillo (Arm) [51, 52]. We found that due to the knockdown of Atg6, the major components of the ZA were seriously mislocalized and accumulated in small intracellular compartments in the apical region (Figures 10(c)–10(e)). As Fmi was shown to regulate planar cell polarity [51], rhodamine-phalloidin staining was used to examine the condition of the wing hairs in Atg6 RNAi cells. 32 hours after pupal formation the control wing hairs were well-developed and very regularly oriented towards the distal end of the wing (Figure 10(a)). In contrast, the wing hairs were poorly developed or completely absent in the Atg6 RNAi region of the wing (Figure 10(b)). Similar to Atg6, UVRAG RNAi also altered the localization of Arm and disoriented the pattern of the wing hairs, whilst Atg14 RNAi had no noticeable effect on these parameters (Figures 11(a)–11(d)).

#### 3.4.2. Atg6 and UVRAG Regulate the Localization Of Basolateral Membrane Proteins

Next, the localization of basolateral membrane proteins was examined in the pupal wings. At this developmental stage, discs large (Dlg) is localized at the apical regions of septate junctions (SJ), whilst the domain of Fasciclin III (Fas III) is expanded throughout the whole SJ [53]. At the same time the level of these proteins is relatively lower in other plasma membrane regions. We found that the RNAi of Atg6 results in the broadening of the Fas III and Dlg containing plasma membrane area, while the detectable amount of these proteins in the SJ is markedly reduced (Figures 10(f) and 10(g)). Likewise, the RNAi of UVRAG also resulted in the disturbance of Fas III localization (Figure 11(f)). Similarly to SJ proteins, the localization of the basal junction (BJ) protein *β*-integrin is also seriously affected by Atg6 or UVRAG knockdown. At this developmental stage the prospective wing intervein cells form large, *β*-integrin containing basal junctions [54], but this process was dramatically blocked when the level of Atg6 or UVRAG was reduced (Figures 10(h) and 11(h)). Since Atg6 or UVRAG RNAi wing intervein cells were unable to form basal junctions between the two epithelial layers of the wing, this suggests that the blistering of RNAi adult wings was due to the lack of proper basal cell adhesion structures. In contrast, Atg14 RNAi had no noticeable effect on the localization of basolateral membrane proteins (Figures 11(e) and 11(g)).

Collectively, our results demonstrate that the UVRAG and Atg6 containing PI3K (III) complex II is essential for the proper localization of membrane proteins and is required for the establishment of epithelial cell polarity, unlike the complex containing Atg14.

## 4. Discussion

Although the key role of Atg6 in mediating autophagy is obvious [8, 9, 33], its role in other processes such as endocytosis is less clear. For example, while several papers dispute the endocytic role of Atg6 [27, 30, 31], others suggest that Atg6 may regulate endocytosis and other processes as well [32, 34]. As many of these studies were carried out in cell culture, the direct participation of Atg6 in other processes in an* in vivo* system had to be demonstrated [7]. For this purpose, we used the well-acclaimed animal model* Drosophila melanogaster,* and we showed that the Atg6, UVRAG, and Vps34-containing PI3K (III) complex is required for multiple cell biological processes.

As Vps34 and Vps15 have been shown to mediate multiple vesicle trafficking events and cell polarity [14–18], we assumed that Atg6, as a component of the PI3K (III) core complex should also mediate such processes. Our data presented here suggest that* Drosophila* Atg6 is an essential endocytosis regulator, as cells lacking Atg6 are unable to produce PI3P and fail to progress endosomes into fully functioning endolysosomes. We showed that this failure ultimately results in the accumulation of abnormal endolysosomal compartments in the apical regions of the cells. These results are in line with the studies on* Drosophila* Vps34 and Vps15 in which animals lacking Vps34 or Vps15 showed a very similar phenotype [15, 17]. Furthermore, our results are very similar to the results obtained in mice lacking Vps15, as the mutant animals accumulate abnormal lysosomes and develop lysosomal storage diseases [55].* Drosophila* Vps15 was shown to regulate the localization of plasma membrane proteins and thus is required for cell polarity [17]. In line with this, we demonstrated that* Drosophila* Atg6 also acts as a cell polarity regulator, since cells lacking Atg6 fail to form junctional complexes and show disturbed basolateral and planar cell polarity as well.

Notch signaling is a very extensively studied pathway [56] in which endocytosis is a key process for Notch activation and also for downregulation [44].* Drosophila* Vps34 mutant cells were shown to accumulate Notch-positive punctae in the eye imaginal disc [15]; therefore it can be assumed that the result of the depletion of Atg6 should be similar. Indeed, we found that Atg6 is required for the endocytic degradation of Notch and Wnt as well; thus possibly Atg6 is required also for downregulation of several other signaling pathways. It has been shown that Notch activation is greatly reduced in mutants that block entry into the early endosome but is enhanced in mutants that increase endosomal retention [44]. This is in line with our observations, as depletion of Atg6 results in slightly enhanced Notch signaling. The data presented above provide further evidence that Atg6 is essential for downregulating this pathway. Mammalian cells in culture accumulate EGFR in small intracellular compartments when the members of PI3K (III) complex are silenced [32]. As in* Drosophila*, Atg6 depleted cells accumulate not only Notch but also Wingless; we assume that Atg6 as a component of PI3K (III) is essential for regulating several important signaling pathways by degrading the endocytosed receptors complexed with their ligands, and this function is greatly conserved among eukaryotes.

UVRAG is a tumor suppressor [21], and as a component of PI3K (III) complex II, it has been suggested to regulate autophagy and vesicle trafficking in mammalian cells [12, 23]. An orthologue of UVRAG was identified in* Drosophila* and was shown to control organ rotation by regulating the degradative endocytic traffic of Notch [57]. Based on a large-scale proteomic study, UVRAG likely binds to Atg6 in* Drosophila* as well [58]. As UVRAG has been speculated to form other complexes in mammals besides PI3K (III) complex II such as with class C Vps [12], the question remained open whether UVRAG regulates Notch signaling as a member of PI3K (III) complex II or this function of UVRAG is independent from this complex. We found that the phenotype of UVRAG RNAi is similar to the phenotype of Atg6 RNAi, as the depletion of either results in enhanced Notch signaling due to the accumulation of Notch. This suggests that Atg6 is responsible for regulating endocytosis and thus Notch and other signaling pathways as well, as a central component of UVRAG-containing PI3K (III) complex II. This presumption is supported by our observation that cells lacking UVRAG accumulate abnormal endolysosomal compartments in a very similar manner to Atg6 RNAi cells. Similarly, in UVRAG depleted cells the selective PI3P marker GFP-2XFYVE is also unable to associate with endosomal membranes, indicating a failure of PI3P production. This raised the possibility that, in other autophagy-independent processes mediated by an Atg6, the PI3K complex II is the key player. Indeed, we found that UVRAG depleted wing cells develop similar polarity defects as Atg6 RNAi cells.

Atg14/Barkor was identified as the mammalian autophagy-specific factor for Beclin 1 and PI3K (III) [19] and is a component of PI3K (III) complex I [11]. In mammalian cells Atg14 is required to recruit PI3K (III) to the formation site of autophagosomes [22]. Although Atg14 is considered to be autophagy specific, there are results showing that Atg14 may also participate in the regulation of endocytosis [26]. The* Drosophila* orthologue of Atg14 was shown to be also essential for autophagy in the fat body [39, 42], but the participation of Atg14 in other PI3K (III) mediated processes remained unclear. We found that although depletion of Atg14 causes very serious malformations in the wing, this effect is neither the consequence of altered cell polarity nor the consequence of endocytosis defects. Atg14 RNAi wing cells develop normal cellular junctions, their endosomal compartments did not differ from control cells, and the ultrastructure of Atg14 depleted pupal wing cells appeared to be normal. Notch signaling and Notch and Wingless localization were completely identical to controls, and cells lacking Atg14 were able to produce PI3P as the distribution and localization of PI3P marker GFP-2XFYVE was similar to wild type cells. Our results suggest that Atg14 may not be required for other PI3K (III) mediated processes other than autophagy in* Drosophila *wing.

## 5. Conclusions

**Figure 12:**
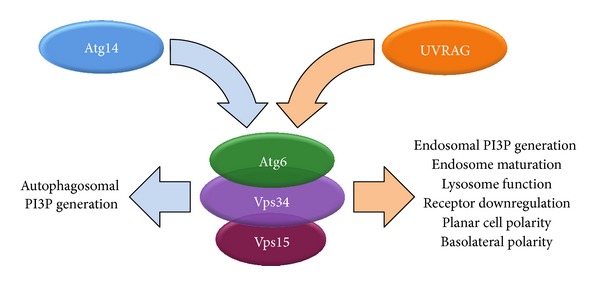
Schematic representation summarizing the multiple roles of the two PI3K (III) complexes in* Drosophila*. See text for details.

Our data presented here suggest that in* Drosophila*, an UVRAG-containing PI3K (III) complex II acts as an essential regulator of endocytosis, membrane trafficking, and is required for downregulating several signaling pathways (Figure 12). Due to these functions, Atg6 is indispensable for proper organ development and cannot be considered as an exclusive autophagy related protein. We propose that the UVRAG-containing PI3K (III) complex II may act as a tumor suppressor, and such a role of Beclin 1 may be masked by its essential function during autophagy, as established cancer cells often depend on autophagy [29, 59].

## Supplementary Material

Fly stocks used in this study are listed in Table S1.
Antibodies used in this study are listed in Table S2.
The details and exact *P* values of statistical analyses are listed in Table S3.
The genotypes of the examined animals are listed in Table S4.
Figure S1: Impaired endocytic degradation of NICD and Delta in Atg6 depleted cells. Imaginal wing discs expressing Atg6 dsRNA by enGal4 were immunostained against (a) the intracellular domain of Notch (NICD) and (c) Delta. The region of RNAi is marked by the coexpression of GFP and the borderline of the region is indicated with a dashed white line. (a,c) In Atg6 RNAi cells both NICD and Delta accumulated in small intracellular dots. (d,b) Quantification of a,c. On box plots, bars show the data ranging between the upper and lower quartiles; median of the signal covered areas is indicated as a horizontal black line within the box. Whiskers plot the smallest and largest observations, while dots and asterisks indicate outliers. NS means *P*>0.05, ∗∗∗ means *P*<0.001. For details and exact p values of statistical analyses see Table S3. For genotype see Table S4. Scale bars represent 50 **μ**m.
Figure S2: The penetrance of the wing phenotype of RNAi lines. Cross indicates increased lethality. See Figure 9 for phenotypes.
Figure S3: Cell death in Atg14 RNAi wing discs. (a-d) Imaginal wing discs expressing RNAi in the wing pouch by Bx^MS1094Gal4^ were immunostained against Wnt. In contrast to (a) controls, in the (b) Atg14 RNAi wing disc numerous cleaved Caspase-3 positive picnotic cells could be observed in the wing pouch (arrows). RNAi of (c) Atg6 or (d) UVRAG did not result in the appearance of cleaved Caspase-3 positive cells in the wing disc. (e-h) TUNEL assay in wing discs. Compared to (e) control or (g) Atg6 RNAi or (h) UVRAG RNAi discs, the wing pouch of (f) Atg14 RNAi discs contained numerous TUNEL-positive cells (arrows), indicating apoptosis. For genotypes see Table S4. Scale bars represent 50 **μ**m.
