# Supplementary material for: Atg6/UVRAG/Vps34-Containing Lipid Kinase Complex Is Required for Receptor Downregulation through Endolysosomal Degradation and Epithelial Polarity during Drosophila Wing Development
Source: Biomed Res Int. 2014 May 21;2014:851349. doi: 10.1155/2014/851349 (PMC4074780; doi:10.1155/2014/851349)
Supplement: Supplementary file 1 — Fly stocks used in this study are listed in Table S1. Antibodies used in this study are listed in Table S2. The details and exact P values of statistical analyses are listed in Table S3. The genotypes of the examined animals are listed in Table S4. Figure S1: Impaired endocytic degradation of NICD and Delta in Atg6 depleted cells. Imaginal wing discs expressing Atg6 dsRNA by enGal4 were immunostained against (a) the intracellular domain of Notch (NICD) and (c) Delta. The region of RNAi is marked by the coexpression of GFP and the borderline of the region is indicated with a dashed white line. (a,c) In Atg6 RNAi cells both NICD and Delta accumulated in small intracellular dots. (d,b) Quantification of a,c. On box plots, bars show the data ranging between the upper and lower quartiles; median of the signal covered areas is indicated as a horizontal black line within the box. Whiskers plot the smallest and largest observations, while dots and asterisks indicate outliers. NS means P>0.05, ∗∗∗ means P<0.001. For details and exact p values of statistical analyses see Table S3. For genotype see Table S4. Scale bars represent 50 μm. Figure S2: The penetrance of the wing phenotype of RNAi lines. Cross indicates increased lethality. See Figure 9 for phenotypes. Figure S3: Cell death in Atg14 RNAi wing discs. (a-d) Imaginal wing discs expressing RNAi in the wing pouch by BxMS1094Gal4 were immunostained against Wnt. In contrast to (a) controls, in the (b) Atg14 RNAi wing disc numerous cleaved Caspase-3 positive picnotic cells could be observed in the wing pouch (arrows). RNAi of (c) Atg6 or (d) UVRAG did not result in the appearance of cleaved Caspase-3 positive cells in the wing disc. (e-h) TUNEL assay in wing discs. Compared to (e) control or (g) Atg6 RNAi or (h) UVRAG RNAi discs, the wing pouch of (f) Atg14 RNAi discs contained numerous TUNEL-positive cells (arrows), indicating apoptosis. For genotypes see Table S4. Scale bars represent 50 μm. [file 851349.f1.pdf]

Table S1. Fly stocks used in this study. (BDSC:Bloomington Drosophila Stock Center, VDRC:Vienna Drosophila RNAi Center)

Stock name	Source [reference]	Stock ID
$Atg6^{1}$	EH. Baechrecke [1]	
UAS-Atg6-myc	EH. Baechrecke [1]	
UAS-GFP-2xFYVE	M. Gonzalez-Gaitan [2]	
tubRab4-YFP	S. Eaton [3]	
tubRab11-YFP	S. Eaton [3]	
tubRab5-CFP, tubRab7-YFP	T. Klein [4]	
UAS-Lamp1-GFP	H. Krämer [5]	
tubLamp1-GFP	H. Krämer [5]	
CathD-mCherry	G. Juhasz [6]	
ptcGal4,UAS-myc,UAS-mCherry-Atg8	G. Juhasz [7]	
MsGal4	BDSC	8860
MsGal4;UAS-Dcr-2	BDSC	25706
UAS-Atg6-RNAi	BDSC	28060
UAS-UVRAG-RNAi	BDSC	34368
UAS-Atg14-RNAi	VDRC	108559
UAS-Vps34-RNAi	VDRC	100296
UAS-Vps15-RNAi	BDSC	34092
UAS-Atg8a-RNAi	BDSC	28989
UAS-N	BDSC	26820
UAS-Hrp-CD2	BDSC	8763
UAS-Clc-GFP	BDSC	7107
UAS-FLP	BDSC	4539
FRT82B,ubiGFP,Minute($RpS3^{Plac92}$)	BDSC	5627
FRT82B,ubiGFP	BDSC	32655
FRT82B	BDSC	2035
hsFLP	BDSC	8862
UAS-Dcr-2;enGal4,UAS-EGFP	BDSC	25752
enGal4	BDSC	30564
enGal4,RFP	BDSC	30557
UAS-Dcr-2;enGal4,UAS-myrRFP,NRE-EGFP	BDSC	30730

Table S2. Antibodies used in this study. (DSHB:Developmental Studies Hybridoma Bank, IHC: immunohistochemistry, IEM: immunoelectron microscopy, TA:traffic assay)

Antibody	Application and dilution	Source
mouse anti-Flamingo	IHC: 1:50	DSHB: Flamingo #74
mouse anti-Armadillo	IHC: 1:100	DSHB: N2 7A1 ARMADILLO
mouse anti-Fasciclin III	IHC: 1:100	DSHB: 7G10 anti-Fasciclin III
rat anti-Cadherin	IHC: 1:100	DSHB: DCAD2
mouse anti-Discs large	IHC: 1:100	DSHB: 4F3 anti-discs large
mouse anti-β-integrin	IHC: 1:80	DSHB: CF.6G11
rabbit anti-GFP	IHC: 1:800, IEM: 1:100	Abcam: ab290-50
mouse anti-Notch extracellular domain	IHC: 1:100 TA: 1:25	DSHB: C458.2H
mouse anti-Notch intracellular domain	IHC: 1:100	DSHB: C17.9C6
mouse anti-Delta extracellular domain	IHC: 1:100	DSHB: C594.9B
mouse anti-Wingless	IHC: 1:100	DSHB: 4D4
rabbit anti-p62	IHC: 1:2000	G.Juhasz [8]
rat anti-mCherry	IHC: 1:300	G. Juhasz [7]
rabbit anti-Cleaved Caspase-3	IHC: 1:300	Cell Signaling Technology®: #9661
Sheep anti-Digoxigenin-AP conjugate	TUNEL: 1:4000	Roche: 11093274910
DyLight®594 horse anti-Mouse	IHC: 1:500	Vector: Dl-2594
DyLight®594 goat anti-Rabbit	IHC: 1:500	Vector: Dl-1594
Alexa Fluor®488 goat anti-Mouse	IHC: 1:500	Invitrogen: A11001
Alexa Fluor®488 goat anti-Rabbit	IHC: 1:500	Invitrogen: A11008
Alexa Fluor®568 goat anti-Rat	IHC: 1:500	Invitrogen: A11077
18nm gold-conjugated goat anti-Rabbit	IEM: 1:100	Jackson ImmunoResearch: 111-215-144

Table S3. Statistics

Figure	Group	N	Mean	SD	P-value	Test and post hoc
Fig. 2a-e GFP-FYVE	control	30	8.2510	3.0501	P<0.0001	Kruskal-Wallis test (ctrl-Atg6: P<0.0001, ctrl-UVRAG: P<0.0001, ctrl-Atg14: P=1.0000, Atg6-UVRAG: P=1.0000, Atg6-Atg14: P<0.0001, UVRAG-Atg14: P<0.0001
	Atg6 RNAi	30	0.6510	1.0100		
	UVRAG RNAi	30	0.7843	0.9453		
	Atg14 RNAi	30	8.5140	4.2216		
Fig. 2f-h Atg6,Clc	control	30	7.5703	3.4941	P=0.1761	Mann-Whitney test
	Atg6 RNAi	30	6.2923	6.7276		
Fig. 2i,j Atg6,Rab4	control	30	9.6607	2.9445	P<0.0001	Wilcoxon's signed ranks test
	Atg6 RNAi	30	29.9700	5.9617		
Fig. 2k,l Atg6,Rab5	control	30	9.1597	4.4263	P<0.0001	Wilcoxon's signed ranks test
	Atg6 RNAi	30	22.5687	7.5319		
Fig. 2m,n Atg6,Rab7	control	30	7.4573	3.6079	P<0.0001	Wilcoxon's signed ranks test
	Atg6 RNAi	30	20.3950	6.5058		
Fig. 2o,p Atg6,Rab11	control	30	0.6627	0.8570	P=0.1494	Wilcoxon's signed ranks test
	Atg6 RNAi	30	1.8077	2.4607		
Fig. 2q,r Atg6,Lamp1	control	30	0.9523	1.0936	P<0.0001	Wilcoxon's signed ranks test
	Atg6 RNAi	30	13.6137	7.4301		
Fig. 2s,t Atg6,CathD	control	30	3.1570	1.7227	P<0.0001	Wilcoxon's signed ranks test
	Atg6 RNAi	30	15.3590	7.6345		
Fig. 2u,v UVRAG,Lamp1	control	30	0.5913	0.5869	P<0.0001	Wilcoxon's signed ranks test
	UVRAG RNAi	30	6.8510	4.3742		
Fig. 2w,x Atg14,Lamp1	control	30	1.0047	0.6866	P=0.4662	Wilcoxon's signed ranks test
	Atg14 RNAi	30	0.8627	0.7680		
Fig. 5e,f Atg6,p62	control	30	0.5380	0.1205	P<0.0001	Wilcoxon's signed ranks test
	Atg6 RNAi	30	5.2780	0.2916		
Fig. 5g,h Atg14,p62	control	30	0.7723	0.1216	P<0.0001	Wilcoxon's signed ranks test
	Atg14 RNAi	30	3.3040	0.2311		
Fig. 5i,j UVRAG,p62	control	30	0.8763	0.1612	P=0.9658	Wilcoxon's signed ranks test
	UVRAG RNAi	30	0.9503	0.2771		
Fig. 6a,b Atg6 null,NECD	control	25	1.9696	1.1696	P<0.0001	Wilcoxon's signed ranks test
	Atg6 null mutant	25	16.3632	3.3465		
Fig. 6c,d Atg6,NECD	control	20	0.6615	1.0481	P=0.0001	Wilcoxon's signed ranks test
	Atg6 RNAi	20	14.7090	4.8574		
Fig. 6e,f UVRAG,NECD	control	20	1.0630	1.2620	P=0.0001	Wilcoxon's signed ranks test
	UVRAG RNAi	20	12.6330	5.4172		
Fig. 6g,h Atg14,NECD	control	20	3.4385	3.2858	P=0.0966	Wilcoxon's signed ranks test
	Atg14 RNAi	20	2.2240	1.9584		
Fig. S1a,b Atg6,NICD	control	20	0.5050	0.8977	P=0.0001	Wilcoxon's signed ranks test
	Atg6 RNAi	20	17.2225	4.4057		
Fig. S1c,d Atg6,Delta	control	20	0.6775	1.0024	P=0.0002	Wilcoxon's signed ranks test
	Atg6 RNAi	20	5.6810	4.4954		

Table S4. Genotypes of the animals examined in this study.

Figure	Genotype
Fig. 1 Control	+/+; tubGal4/+
Fig. 1 Atg6 RNAi	UAS-Dcr-2/+; tubGal4/UAS-Atg6-RNAi
Fig. 1 UVRAG RNAi	UAS-Dcr-2/+; tubGal4/UAS-UVRAG-RNAi
Fig. 1 Atg14 RNAi	UAS-Atg14-RNAi /+; tubGal4/+
Fig. 2a	MsGal4/+; UAS-Dcr-2/UAS-GFP-2xFYVE; +/+
Fig. 2b	MsGal4/+; UAS-Dcr-2/UAS-GFP-2xFYVE; UAS-Atg6-RNAi/+
Fig. 2c	MsGal4/+; UAS-Atg14-RNAi/UAS-GFP-2xFYVE,UAS-Dcr-2; +/+
Fig. 2d	MsGal4/+;UAS-GFP-2xFYVE/+; UAS-UVRAG-RNAi/+; +/+
Fig. 2f	MsGal4/+; UAS-Dcr-2/UAS-Clc-GFP;+/+
Fig. 2g	MsGal4/+; UAS-Dcr-2/UAS-Clc-GFP; UAS-Atg6-RNAi/+
Fig. 2i	enGal4,RFP/UAS-Dcr-2; UAS-Atg6-RNAi/tubRab4-YFP
Fig. 2k	enGal4,RFP/UAS-Dcr-2; UAS-Atg6-RNAi/tubRab5-CFP
Fig. 2m	enGal4,RFP/UAS-Dcr-2; UAS-Atg6-RNAi/tubRab7-YFP
Fig. 2o	enGal4,RFP/UAS-Dcr-2; UAS-Atg6-RNAi/tubRab11-YFP
Fig. 2q	enGal4,RFP/tubLamp1-GFP; UAS-Atg6-RNAi/UAS-Dcr-2
Fig. 2s	UAS-Dcr-2/+; enGal4,UAS-EGFP/CathDmCherry; UAS-Atg6-RNAi/+
Fig. 2u	enGal4,RFP/tubLamp1-GFP; UAS-UVRAG-RNAi/UAS-Dcr-2
Fig. 2w	enGal4,RFP/tubLamp1-GFP,UAS-Atg14-RNAi; UAS-Dcr-2/+
Fig. 3a	MsGal4/+; UAS-Dcr-2/+; +/+
Fig. 3b	MsGal4/+; UAS-Dcr-2/+; UAS-Atg6-RNAi/+
Fig. 3c	MsGal4/+; UAS-FLP/+; FRT82bAtg6^{1}/FRT82b,ubiGFP,Minute(RpS3^{Plac92})
Fig. 3d	MsGal4/+; UAS-Dcr-2/+; UAS-Atg8a-RNAi/+
Fig. 3e	MsGal4/+; UAS-Dcr-2/+; UAS-UVRAG-RNAi/+
Fig. 3f	MsGal4/+; UAS-Dcr-2/UAS-Atg14-RNAi; +/+
Fig. 4a,b	MsGal4/+; UAS-Dcr-2/UAS-Lamp1-GFP; UAS-Atg6-RNAi/+
Fig. 4c	MsGal4/+; UAS-Dcr-2/+; +/+
Fig. 4d-f	MsGal4/+; UAS-Dcr-2/+; UAS-Atg6-RNAi/+
Fig. 4g	MsGal4/+; UAS-Dcr-2/UAS-HRP-CD2; +/+
Fig. 4h	MsGal4/+; UAS-Dcr-2/UAS-Hrp-CD2; UAS-Atg6-RNAi/+
Fig. 5a	ptc-Gal4, UAS-mCherry-Atg8a, UAS-Myc/+; +/+
Fig. 5b	ptc-Gal4, UAS-mCherry-Atg8a, UAS-Myc/+; UAS-Atg6-RNAi /+
Fig. 5c	ptc-Gal4, UAS-mCherry-Atg8a, UAS-Myc/ UAS-Atg14-RNAi; +/+
Fig. 5d	ptc-Gal4, UAS-mCherry-Atg8a, UAS-Myc/+; UAS-UVRAG-RNAi /+
Fig. 5e	UAS-Dcr-2/+; enGal4,UAS-EGFP/+; UAS-Atg6-RNAi/+
Fig. 5g	UAS-Dcr-2/+;enGal4,UAS-EGFP/UAS-Atg14-RNAi;+/+
Fig. 5i	UAS-Dcr-2/+; enGal4,UAS-EGFP/+; UAS-UVRAG-RNAi/+
Fig. 6a	hsFLP/+; +/+; FRT82bAtg61/FRT82b,ubiGFP
Fig. 6c	UAS-Dcr-2/+; enGal4,UAS-EGFP/+;UAS-Atg6-RNAi/+
Fig. 6e	UAS-Dcr-2/+; enGal4,UAS-EGFP/+; UAS-UVRAG-RNAi/+
Fig. 6g	UAS-Dcr-2/+; enGal4,UAS-EGFP/UAS-Atg14-RNAi; +/+
Fig. 6i	enGal4/UAS-Lamp1-GFP; UAS-Atg6-RNAi/UAS-Dcr-2
Fig. 7a	UAS-Dcr-2/+; enGal4, UAS-myr-mRFP,NRE-EGFP/+; UAS-Atg6-RNAi/+

Fig. 7b	UAS-Dcr-2/+; enGal4, UAS-myr-mRFP,NRE-EGFP/+; UAS-N/+
Fig. 7c	enGa4,UAS-myr-mRFP,NRE-EGFP/UAS-Dcr-2;UAS-Atg6-RNAi/UAS-N
Fig. 7d	UAS-Dcr-2/+;enGal4,UAS-myr-mRFP,NRE-EGFP/+; UAS-UVRAG-RNAi/+
Fig. 7e	UAS-Dcr-2/+; enGal4, UAS-myr-mRFP,NRE-EGFP/UAS-Atg14-RNAi; +/+
Fig. 7f	UAS-Dcr-2/+; enGal4, UAS-myr-mRFP,NRE-EGFP/+; +/+
Fig. 8a	UAS-Dcr-2/+; enGal4,UAS-EGFP/+; UAS-Atg6-RNAi/+
Fig. 8b	UAS-Dcr-2/+; enGal4,UAS-EGFP/+; UAS-UVRAG-RNAi/+
Fig. 8c	UAS-Dcr-2/+;enGal4,UAS-EGFP/UAS-Atg14-RNAi;+/+
Fig. 9a,f	MsGal4/+; UAS-Dcr-2/+; +/+
Fig. 9b-d	MsGal4/+; UAS-Dcr-2/+; UAS-Atg6-RNAi/+
Fig. 9e	MsGal4/+; UAS-Dcr-2/+; UAS-Vps15-RNAi/+
Fig. 9g,h	MsGal4/+; UAS-Dcr-2/+; UAS-UVRAG-RNAi/+
Fig. 9i	MsGal4/+; UAS-Dcr-2/UAS-Vps34-RNAi; +/+
Fig. 9j	MsGal4/+;UAS-Dcr-2/UAS-Atg14-RNAi;+/+
Fig. 9k	MsGal4/y; UAS-FLP/+; FRT82b,ry^{506}/FRT82b,ubiGFP,Minute($RpS3^{Plac92}$)
Fig. 9l	MsGal4/y; UAS-FLP/+; FRT82b$Atg6^{1}$/FRT82b,ubiGFP,Minute($RpS3^{Plac92}$)
Fig. 9m	MsGal4/y; UAS-FLP/UAS-Atg6-myc; FRT82b$Atg6^{1}$/FRT82b,ubiGFP,Minute($RpS3^{Plac92}$)
Fig. 10a-h	UAS-Dcr-2/+; enGal4,UAS-EGFP/+; UAS-Atg6-RNAi/+
Fig. 11a,c,e,g	UAS-Dcr-2/+;enGal4,UAS-EGFP/UAS-Atg14-RNAi;+/+
Fig. 11b,d,f,h	UAS-Dcr-2/+; enGal4,UAS-EGFP/+; UAS-UVRAG-RNAi/+
Fig. S1a,c	UAS-Dcr-2/+; enGal4,UAS-EGFP/+; UAS-Atg6-RNAi/+
Fig. S3a,e	MsGal4/+; UAS-Dcr-2/+; +/+
Fig. S3b,f	MsGal4/+;UAS-Dcr-2/UAS-Atg14-RNAi;+/+
Fig. S3c,g	MsGal4/+; UAS-Dcr-2/+; UAS-Atg6-RNAi/+
Fig. S3d,h	MsGal4/+; UAS-Dcr-2/+; UAS-UVRAG-RNAi/+

Supplementary references:

1. Shravage BV, Hill JH, Powers CM, Wu L, Baehrecke EH: **Atg6 is required for multiple vesicle trafficking pathways and hematopoiesis in Drosophila**. *Development* 2013, **140**:1321–1329.

2. Wucherpfennig T, Wilsch-Bräuninger M, González-Gaitán M: **Role of Drosophila Rab5 during endosomal trafficking at the synapse and evoked neurotransmitter release**. *The Journal of cell biology* 2003, **161**:609–624.

3. Marois E, Mahmoud A, Eaton S: **The endocytic pathway and formation of the Wingless morphogen gradient**. *Development* 2006, **133**:307–317.

4. Troost T, Jaeckel S, Ohlenhard N, Klein T: **The tumour suppressor Lethal (2) giant discs is required for the function of the ESCRT-III component Shrub/CHMP4**. *Journal of cell science* 2012, **125**:763–776.

5. Pulipparacharuvil S, Akbar MA, Ray S, Sevrioukov EA, Haberman AS, Rohrer J, Krämer H: **Drosophila Vps16A is required for trafficking to lysosomes and biogenesis of pigment granules**. *Journal of cell*

science 2005, **118**:3663–3673.

6. Takáts S, Pircs K, Nagy P, Varga Á, Kárpáti M, Hegedűs K, Kramer H, Kovács AL, Sass M, Juhász G: **Interaction of the HOPS complex with Syntaxin 17 mediates autophagosome clearance in Drosophila**. *Molecular biology of the cell* 2014:mbc–E13.

7. Nagy P, Varga Á, Pircs K, Hegedűs K, Juhász G: **Myc-driven overgrowth requires unfolded protein response-mediated induction of autophagy and antioxidant responses in Drosophila melanogaster**. *PLoS genetics* 2013, **9**:e1003664.

8. Pircs K, Nagy P, Varga A, Venkei Z, Erdi B, Hegedus K, Juhasz G: **Advantages and limitations of different p62-based assays for estimating autophagic activity in Drosophila**. *PloS one* 2012, **7**:e44214.
