# Supplementary figures and images for: Atg6/UVRAG/Vps34-Containing Lipid Kinase Complex Is Required for Receptor Downregulation through Endolysosomal Degradation and Epithelial Polarity during Drosophila Wing Development
Source: Biomed Res Int. 2014 May 21;2014:851349. doi: 10.1155/2014/851349 (PMC4074780; doi:10.1155/2014/851349)

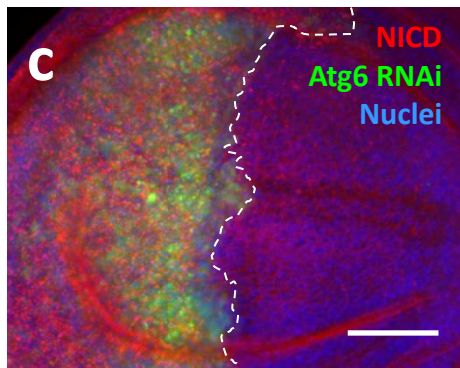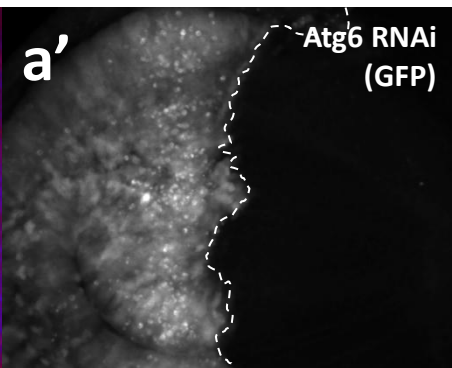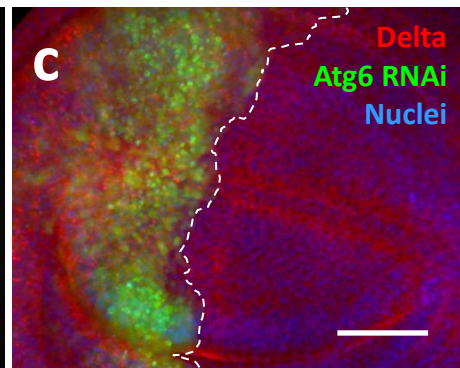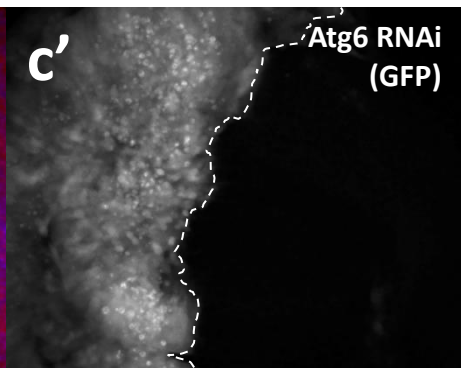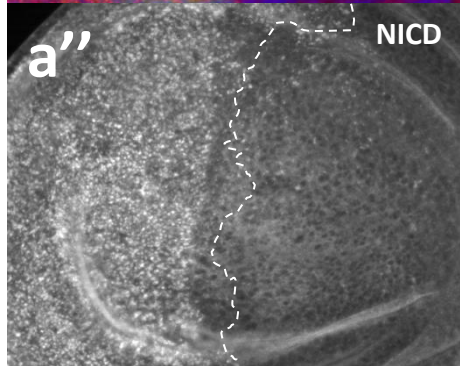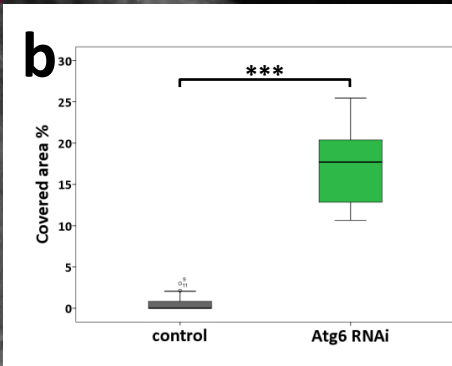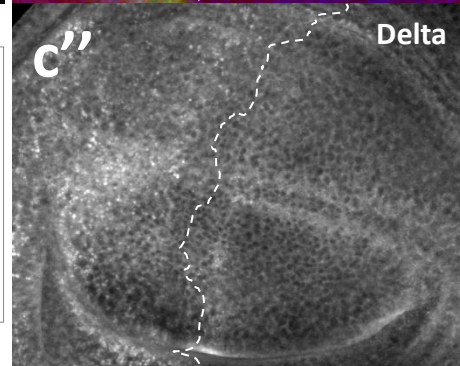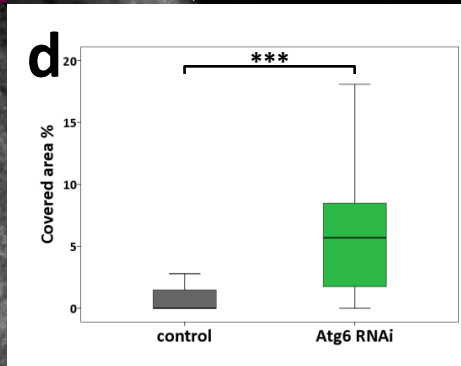

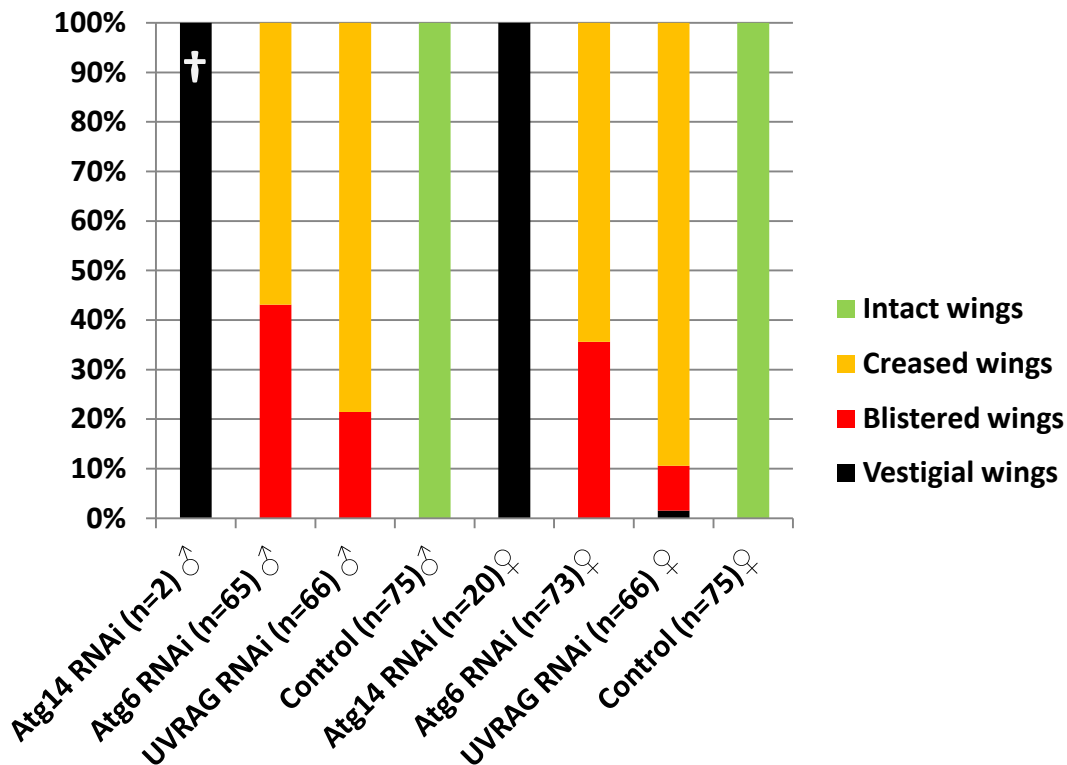

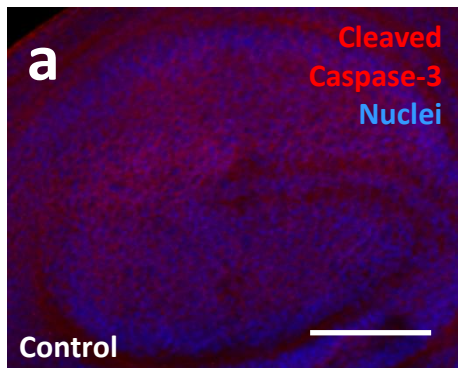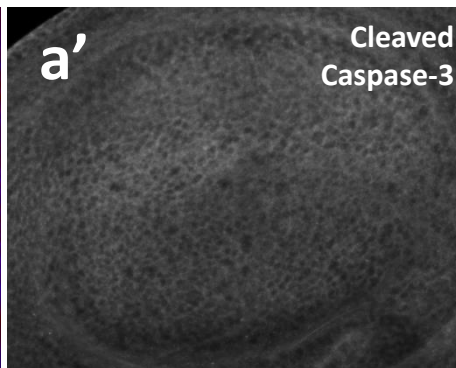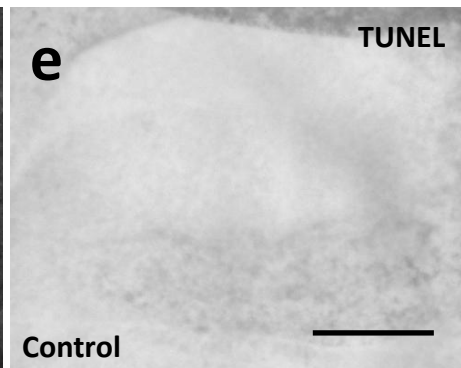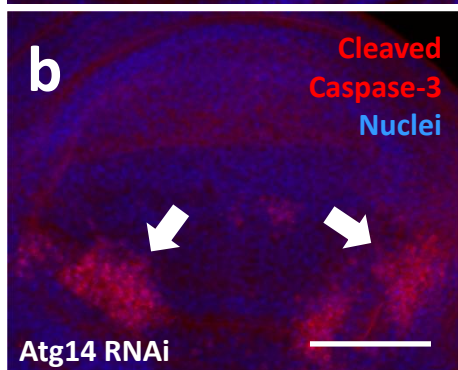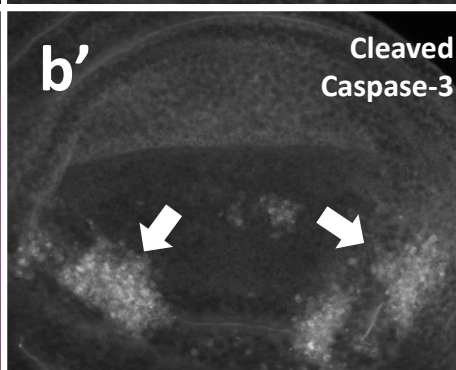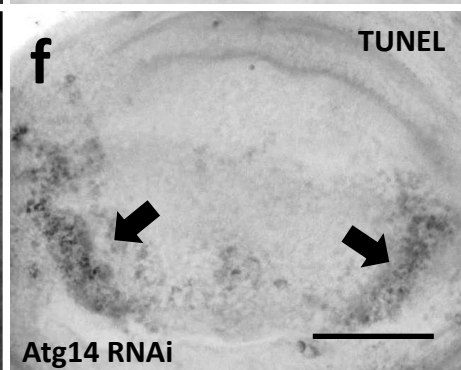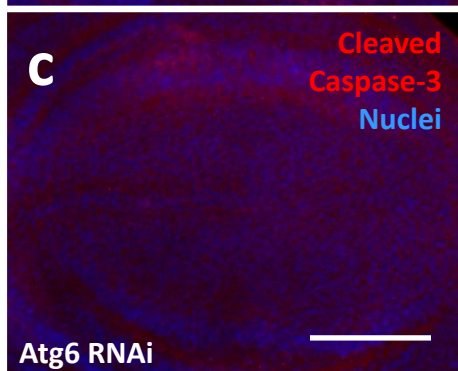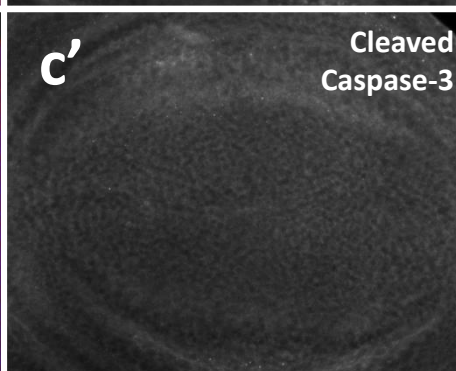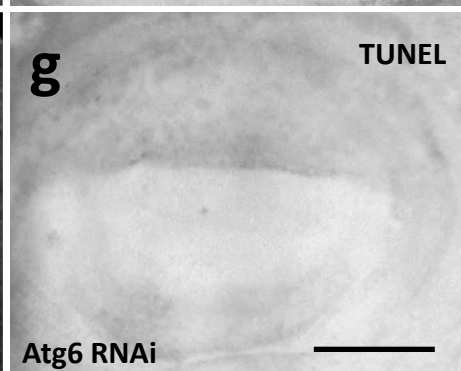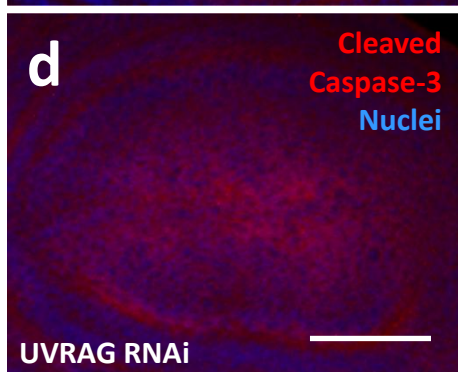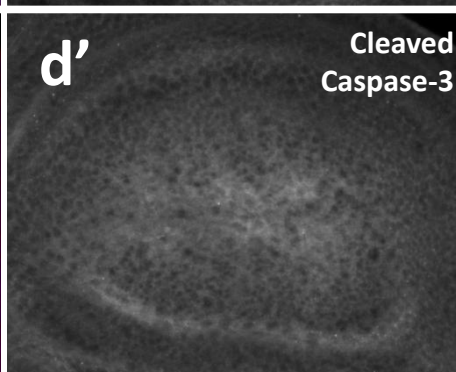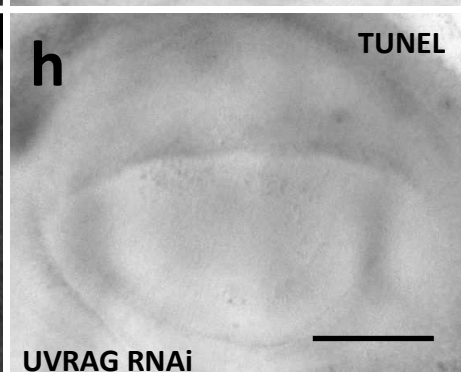

Supplement: Supplementary file 2 [file 851349.f2.pdf]
